# A Strain-Data-Driven Factor-Wise Inverse Identification Approach for Blown-Sand Erosion Parameters of GFRP Strips

**DOI:** 10.3390/ma19173713

**Published:** 2026-08-31

**Authors:** Bingyu Han, Jiayi Yang, Shuai Hao, Yufeng Liu, Xueqiong Zhou, Wenhao Feng

**Affiliations:** 1 Transportation Institute, Inner Mongolia University, Hohhot 010070, China; byhan@imu.edu.cn (B.H.); 311980385@imu.edu.cn (X.Z.); 2 Inner Mongolia Key Laboratory of Structural Testing for Colleges and Universities, Inner Mongolia University, Hohhot 010070, China; 3 School of Civil Engineering, Inner Mongolia University of Technology, Hohhot 010051, China; 15849573915@163.com (J.Y.); haoshuai677@163.com (S.H.); 15661160276@163.com (Y.L.)

**Keywords:** blown-sand erosion, GFRP strips, residual mechanical properties, micro-analysis, inverse model, engineering protection

## Abstract

Glass fiber-reinforced polymer (GFRP) is widely used in wind power, construction, and aerospace for its superior properties. However, studies on its blown-sand erosion degradation and prediction remain limited. This study investigates the degradation and prediction of blown-sand erosion behavior in GFRP strips using a one-factor-at-a-time (OFAT) experimental design. Simulated experiments quantify the effects of factors such as erosion angle, velocity, sand flow rate, and erosion time on mechanical behavior. Results show that epoxy-layer deformation and internal fiber fracture increase with erosion angle and velocity. The tensile strength decreased by approximately 16.8% at an erosion angle of 90° when the velocity, sand flow rate, and erosion time were fixed at 26 m/s, 55 g/min, and 30 min, respectively. At an erosion velocity of 31 m/s, with the erosion angle, sand flow rate, and erosion time fixed at 90°, 55 g/min, and 30 min, respectively, the strength reduction reached 28%. Under a 45 g/min sand flow rate, the strength reduction reached 6%, while extending the erosion time to 50 min led to a decrease of 35%. Furthermore, a particle swarm optimization (PSO)-assisted, interpolation-based inverse identification model was established to back-calculate erosion parameters from measured strain fields. The mean in-sample reconstruction error was approximately 4.2%, whereas leave-one-condition-out validation yielded an overall mean error of 31.8%, with factor-specific errors ranging from 12.8% to 43.8%, indicating limited generalization to unseen conditions. This work elucidates the progression of material degradation from initial damage to severe failure and provides laboratory reference data for understanding the post-erosion residual behavior under the investigated erosion-only conditions.

## 1. Introduction

Glass fiber-reinforced polymer (GFRP) strips have received widespread attention in engineering applications because of their low density, high strength, corrosion resistance, and other favorable properties. In order to better exploit the superiority of each material, hybrid structures combining GFRP fabric and concrete have been developed, and studies have shown that GFRP strips can be used not only as tensile reinforcement but also as fixed formwork [1,2]. It can significantly improve the durability of concrete columns and can be applied to the rehabilitation and performance improvement of in-service highway and railway bridges in the northwest of China. The northwest of China is located in Central Asia, which is one of the world’s four major sandstorm epicenters. The high frequency of sandstorms in the area consequently imposes substantial erosion risks on infrastructure. Blown-sand erosion is mainly an erosive wear process of sand particles, which is one of the main causes of wear of structural materials and can negatively affect the durability of GFRP strips [3,4,5]. The residual mechanical properties of GFRP strips after blown-sand erosion are critically linked to their service life and structural reliability. Therefore, it is essential to investigate how blown-sand erosion influences these properties in order to assess long-term performance, establish predictive models for material degradation, and support the development of more durable composite structures in erosion-prone environments [6].

Based on the gas–solid jet method, an experimental study was carried out on the erosion resistance of GFRP composite material against solid particle erosion, and the results showed that the erosion would cause serious erosion of GFRP composite material [7]. The initial consequence of sand erosion is the deterioration of the epoxy adhesive layer on the surface of the GFRP composite material. This deterioration is the result of the continuous erosion, which subsequently leads to the fracture of the composite material’s internal fibers. The stress concentration phenomenon is of particular concern in the context of localized damage, as it can significantly impact the overall stress performance of GFRP composite materials. This is due to the failure of local colloids and fibers, which can lead to the degradation of the material’s integrity and strength [8,9,10]. A study was conducted to compare the erosion resistance of carbon fiber reinforced polymer (CFRP) and GFRP materials when subjected to the same conditions as particulate matter. The results indicated that GFRP materials demonstrated a higher propensity for damage under identical conditions. In the context of blown-sand environments, the damage degree of GFRP fabrics is influenced by numerous factors, including erosion conditions, the properties of the target material, and the characteristics of particles. Relevant studies have demonstrated that these factors are associated with the residual mechanical properties of GFRP strips [11,12,13]. A comprehensive analysis of the impact of erosion factors reveals that the erosion angle, target material composition, particle size, and temperature exert a significant influence on the erosion results of GFRP materials. It is noteworthy that the erosion velocity exerts the most pronounced impact.

Related studies have investigated the damage mechanisms of GFRP strips under solid particle erosion [14,15,16]. However, research on the residual mechanical properties of eroded GFRP strips remains relatively limited, with most existing work focusing primarily on the observation and evaluation of surface-layer damage. In practical engineering, the residual mechanical properties of GFRP strips after blown-sand erosion are related to their continuous reinforcement and service performance, so it is of great importance to investigate the degradation mechanism of GFRP strips in blown-sand environments and the deterioration mechanism for their engineering durability design. Digital image correlation (DIC) technology has non-contact, full-field deformation measurement, data accuracy, and other outstanding advantages; these advantages make DIC technology become an indispensable tool in modern engineering testing and scientific research. In the field of civil engineering, materials damage and deterioration research is highly favored. The utilization of DIC technology facilitates the acquisition of precise deformation data concerning the fiber material’s response to applied stress. Consequently, it can be deduced that the employment of DIC technology enhances the opportunity to acquire the overall mechanical behavior of GFRP strips subjected to blown-sand erosion [17]. This capacity of DIC technology has prompted numerous scholars to employ it in research endeavors pertaining to the damage of GFRP materials [18,19,20].

In the preceding paper, the damage characteristics of GFRP strips under blown-sand erosion were systematically investigated by experiments, and the correlation law between macroscopic mechanical response and erosion conditions was elucidated. However, it should be noted that the experimental method is subject to inherent limitations. Firstly, the complex coupling effect of wind and sand environment is difficult to fully reproduce within the finite working conditions of the experiment [21]. Secondly, the damage emergence and expansion mechanism at the fine scale is difficult to accurately capture due to the constraints of the experimental observation method [22]. In order to overcome the limitations of experimental studies in complex environment simulation and long-period damage observation, scholars are actively developing multi-scale numerical simulation and data fusion algorithms to reveal the damage evolution law of GFRP from the two dimensions of physical mechanism and parameter correlation.

The finite element method (FEM) and related numerical approaches are regarded as core tools for studying the environmental behavior of composite materials, enabling full-scale simulation that links microscopic damage mechanisms to macroscopic mechanical responses. The following studies illustrate this capability, ranging from the development of damage and failure criteria to the analysis of structural applications. Cen et al. [23] established the Lamb wave dynamic failure criterion, which provides a high-precision prediction method of composite material damage evolution. Yousif et al. [24] conducted a synergistic analysis of GFRP composites under impact loading, combining numerical and experimental methods. The reliability of the coupled multi-physics field simulation was verified. Cheng et al. [25] developed a steel–GFRP–foam composite crashworthiness device, while demonstrating the innovative structural application of GFRP materials under extreme working conditions. Dadras et al. [26] developed nanosilica/nanoclay-reinforced GFRP composites and investigated their mechanical degradation in acidic environments, while employing FEM and artificial neural network (ANN) to predict indentation behavior and immersion effects.

To circumvent the limitations of a single methodology, intelligent optimization algorithms have been increasingly adopted. Nouri et al. [27] and Khan et al. [28] demonstrated the advantages of such algorithms in capturing nonlinear relationships, and the hybrid optimization algorithm of Khan et al. [28] reduced the durability prediction error to 3.8%. Babiker et al. [29] developed a deep-learning–regression hybrid model for accurate prediction of the punching shear strength of GFRP joints. Wu et al. [30] established an acoustic-emission–bond-strength correlation model that offers a new paradigm for structural health monitoring. Karimipour et al. [31] combined evolutionary strategies with artificial neural networks to predict the load-carrying capacity of GFRP-reinforced concrete columns, providing support for multi-objective optimization.

Multi-factor coupling effects have also been widely investigated. Wang et al. [32] revealed the degradation of GFRP–concrete interfacial bond performance in high-temperature environments; Mohanraj et al. [33] optimized the parameters of GFRP hole-making processes; He et al. [34] verified the shear-enhancement effect of GFRP–steel hybrid stirrups; and Brahim et al. [35] proposed a neural network prediction of repair strength. On the experimental validation side, Wei et al. [36] developed a long-pulse thermographic depth-detection technique, Panchagnula et al. [37] established a deep-learning diagnostic model for drilling defects, Fahem et al. [38] proposed an improved Jaya–ANN hybrid algorithm, Wang et al. [39] developed a hygrothermal-aging correlation model, and Ali et al. [40] presented a reliability-assessment method for GFRP deep beams. Together, these studies provide multi-level support for constructing and validating coupled numerical models.

To address the limitations of conventional experimental approaches in characterizing blown-sand erosion damage and its associated mechanical response, this study combines gas–solid erosion tests, digital image correlation (DIC)-based deformation measurements, and microstructural characterization to investigate the residual behavior of GFRP strips under different erosion conditions. A one-factor-at-a-time (OFAT) experimental design is adopted, in which erosion angle, erosion velocity, sand flow rate, and erosion time are varied individually while the remaining factors are maintained at their prescribed baseline values. Based on the resulting factor-wise experimental datasets, separate interpolation relationships are established between each erosion factor and the corresponding strain response of the GFRP strips. Particle swarm optimization (PSO) is then employed to solve the inverse problem for each erosion factor individually by minimizing the difference between the measured and interpolated strain responses. Accordingly, the proposed method is intended as a factor-wise inverse identification approach for investigating the relationship between individual erosion conditions and the post-erosion mechanical response of GFRP strips, rather than as a coupled four-parameter prediction model. The results provide experimental evidence for understanding erosion-induced degradation and a methodological basis for further development of inverse identification approaches for GFRP materials exposed to blown-sand environments.

## 2. Overview of Experiment

### 2.1. Specimen Design

**Figure 1:**
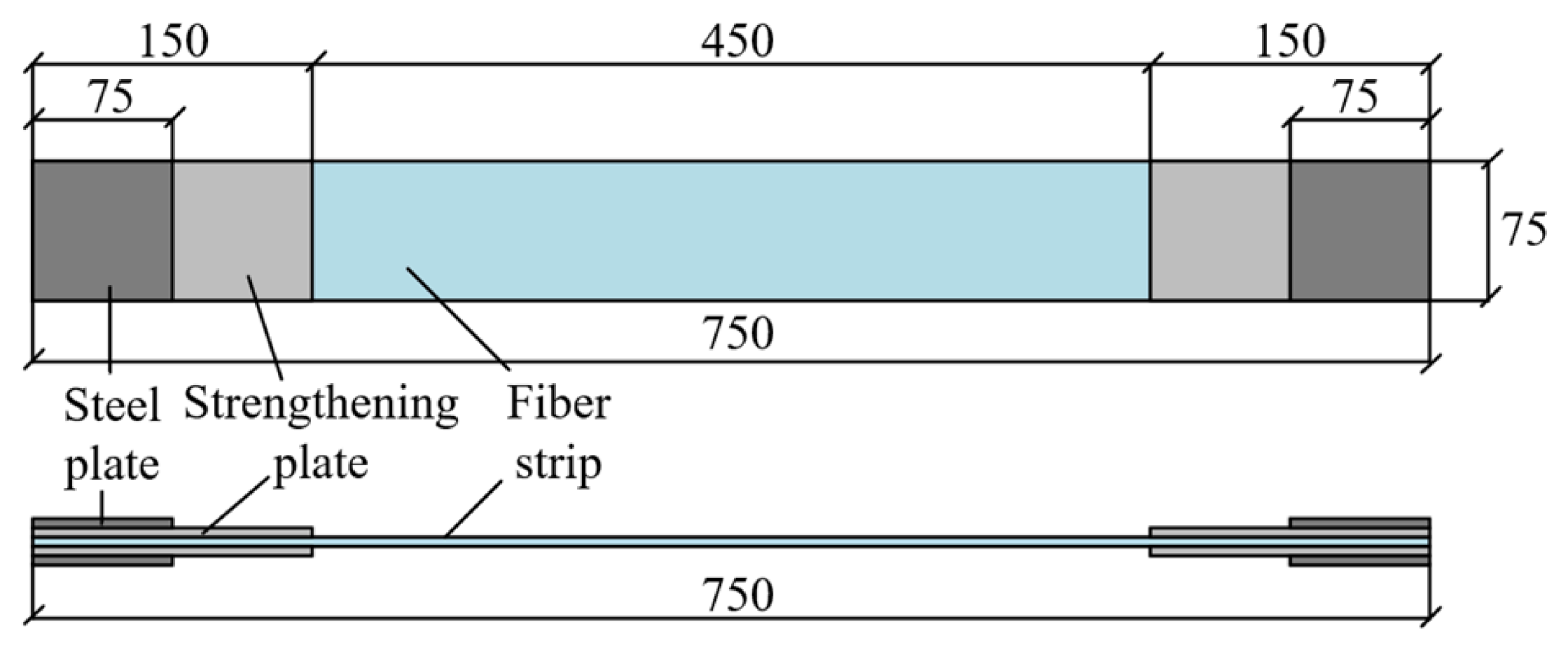
Size of GFRP specimens (size: mm).

**Figure 2:**
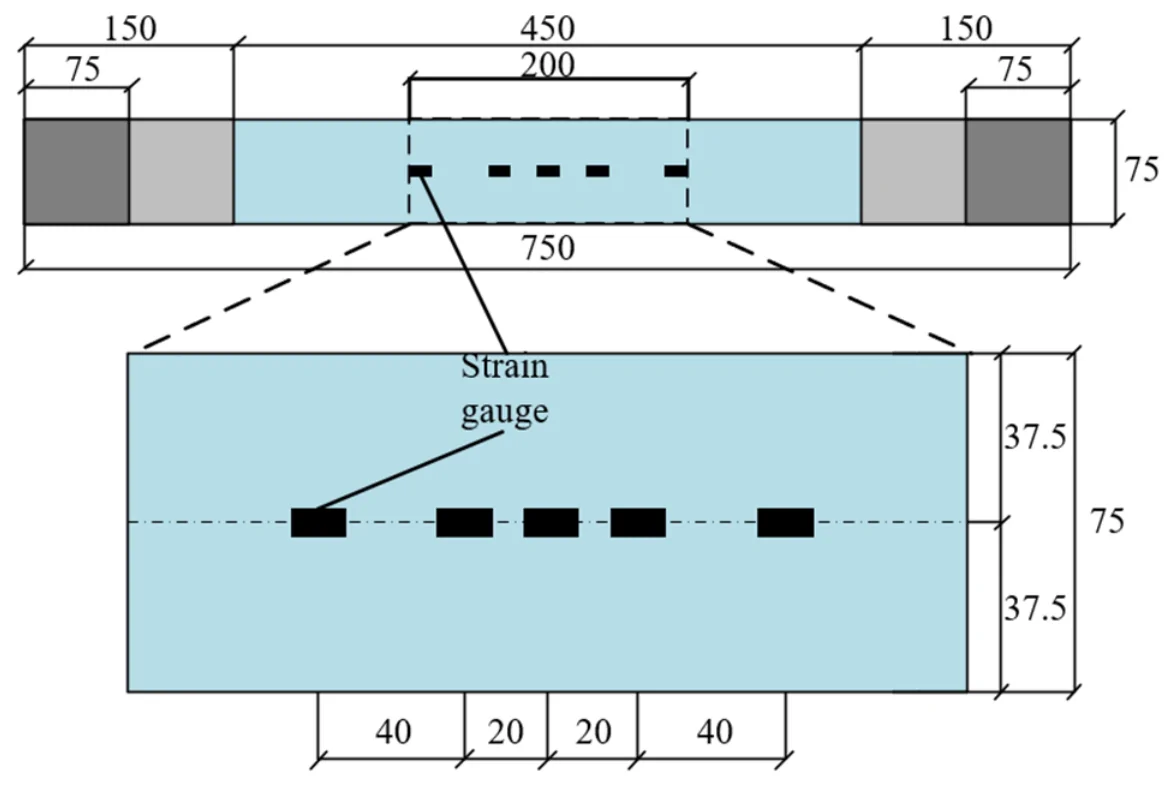
Strain gauge arrangement (size: mm).

The GFRP strips used in the current test were provided by a well-established domestic manufacturer. The fibers were cured and molded using a two-component epoxy resin adhesive. The material performance parameters of the GFRP strips provided by the manufacturer are shown in Table 1. The dimensions and fabrication of the test specimen were determined in accordance with the specifications outlined in GB/T 1447-2005 (Standardization Administration of the People’s Republic of China 2005) and GB/T 3354-2014 (Standardization Administration of the People’s Republic of China 2005). The precise dimensional parameters are illustrated in Figure 1. Subsequent to the fabrication of the specimens, they were subjected to a curing process in a laboratory setting at a temperature of 20 °C for a duration of approximately six hours. This was followed by the blown-sand erosion test. Concurrently, in order to analyze the material deformation information around the erosion damage area, five strain gauges were affixed on the surface of the GFRP strips, which can be found in Figure 2.

### 2.2. Erosion Test Setup

**Figure 3:**
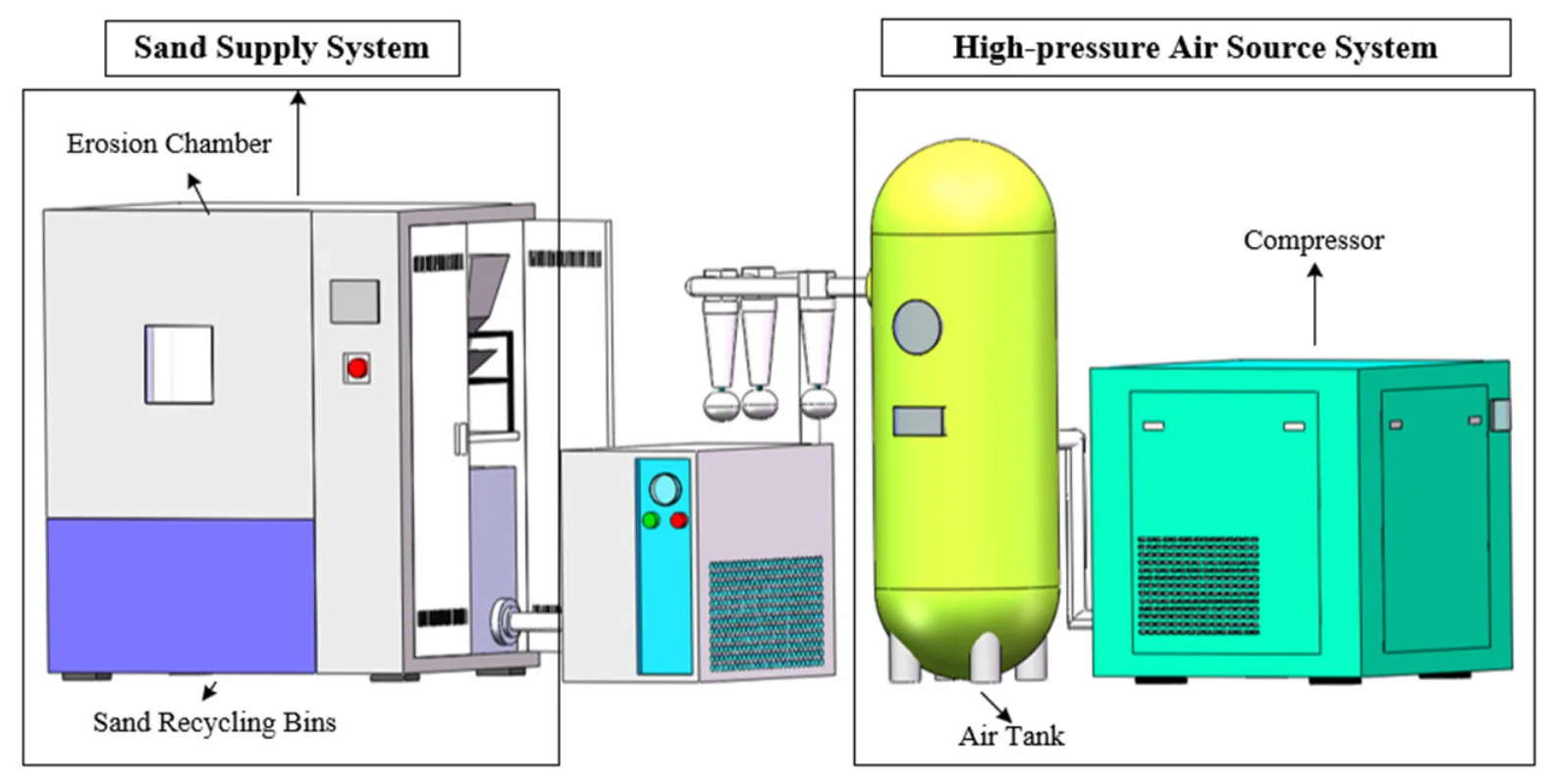
Blown-sand erosion test setup.

The erosion test adopts the airflow sand jet method through a simulated blown-sand environment erosion test system. This method, which has been employed in numerous extant studies, has the capacity to more realistically simulate the actual blown-sand environment. Furthermore, the durability test period is shorter, and it could accurately control the influencing parameters. As illustrated in Figure 3, the blown-sand erosion experimental system under consideration in this paper is composed of the following components: an air compression system, a sand supply system, an erosion system, and other components.

The sand utilized in the test was taken from wind-deposited sand in the Kubuqi Desert, which is located in Inner Mongolia. This sand was subjected to a sieving process to remove impurities, subsequently allowing its application in the erosion test. This region experiences approximately 25–35 days of strong wind per year, with the strongest sandstorms reaching a wind force of 9. The surface sediment of the Kubuqi Desert is dominated by fine and very fine sand, whose combined content exceeds 85%; accordingly, the grain size of the erodent is mainly distributed in the fine to very fine sand range (about 0.075–0.25 mm). The sand particles have a Vickers micro-hardness of about 7750 MPa (Mohs hardness of grade 6) and a particle density of 2.7 g/cm^3^, reflecting the hard, predominantly quartz composition of the natural sand and its strong abrasive capacity against the GFRP surface. In consideration of the prevalence of sandstorms in the northwest of China, the physical properties of solid sand particles, the velocity of blown sand, the sand concentration, and other pertinent factors, as well as a review of existing relevant studies, six erosion angles of 15°, 30°, 45°, 60°, 75°, and 90° were selected as the angle variables (as presented in Table 2). The erosion angle here refers to the impact angle between the sand jet direction and the specimen surface, in accordance with the convention commonly adopted in solid particle erosion studies. Five velocities of 16 m/s, 19 m/s, 23 m/s, 26 m/s, and 31 m/s were determined as the velocity variables, where the reported velocity is the velocity of the sand-laden air jet measured at the nozzle outlet. And five sand flow rates of 15 g/min, 25 g/min, 35 g/min, 45 g/min, and 55 g/min were used as the sand flow rate variables; the sand flow rate denotes the mass of sand fed into the air stream per unit time and is used to characterize the particle loading, with the air supply pressure kept constant so that the sand-to-air ratio increases with the sand flow rate. Moreover, five erosion time of 10 min, 20 min, 30 min, 40 min, and 50 min were chosen as the erosion time. The experimental program consisted of four OFAT test series, with three parallel specimens tested at each parameter level. It should also be noted that in the study of the erosion angle effects, the erosion velocity was 26 m/s, the sand flow rate was 55 g/min, and the erosion time was 30 min. In the study of the erosion velocity effects, the erosion angle was 90°, the sand flow rate was 55 g/min, and the erosion time was 30 min. In the study of sand flow rate, the erosion angle was 90°, the erosion velocity was 26 m/s, and the erosion time was 30 min. In the study of erosion time, the erosion angle was 90°, the erosion velocity was 26 m/s, and the sand flow rate was 55 g/min.

### 2.3. Residual Mechanical Property Testing and Characterization

**Figure 4:**
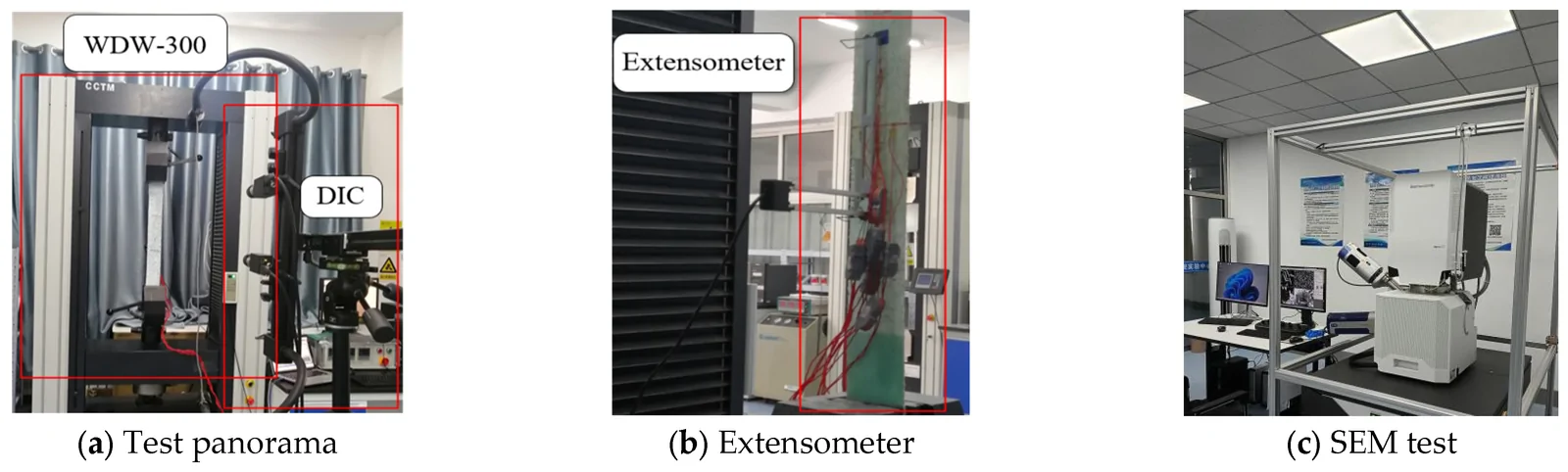
Test setup.

After the GFRP strips had undergone the blown-sand erosion test, their residual mechanical properties were tested, with particular attention to the ultimate tensile strength, tensile elastic modulus, and strain field distribution. No mechanical load was applied to the GFRP strips during the erosion stage; the tensile tests were conducted only after completion of the prescribed erosion exposure. Accordingly, the present tests characterize the post-erosion residual behavior of initially unloaded GFRP strips and do not reproduce simultaneous mechanical loading and erosion or the possible load–erosion interaction occurring in service. Figure 4 shows the test setup for the residual mechanical properties of GFRP strips in which direct tensile testing was performed by a WDW-300 electronic universal materials testing machine; the loading rate was 1 mm/min, and an extensometer was placed in the middle 200 mm of the specimen to observe and record the stiffness of the GFRP strips. The tensile tests of the eroded specimens were carried out following the same standards adopted for specimen preparation, namely GB/T 1447-2005 and GB/T 3354-2014, so that the residual tensile strength and elastic modulus were measured under standardized conditions. A strain collection system was used to obtain strain data in the erosion region of GFRP strips. Full-field deformation was measured using a VIC-3D SR digital image correlation (DIC) system with an image resolution of 1920 × 1200 pixels. A random speckle pattern was spray-painted on the specimen surface, and images were recorded at 1 Hz from a working distance of approximately 1 m and analyzed using VIC-3D 9. The axial strain was calculated as *ε* = Δ*l*/*L*. The nominal strain resolution of the system is 10 με; no separate uncertainty calibration was performed for the present tests. In this paper, in addition to testing the residual mechanical properties of the GFRP strips, the micromorphological observation of the blown-sand erosion damage area was carried out, and the micromorphological examination of the erosion area was performed using a scanning electron microscope (SEM).

## 3. Experimental Results and Analysis

### 3.1. Residual Mechanical Properties

**Figure 5:**
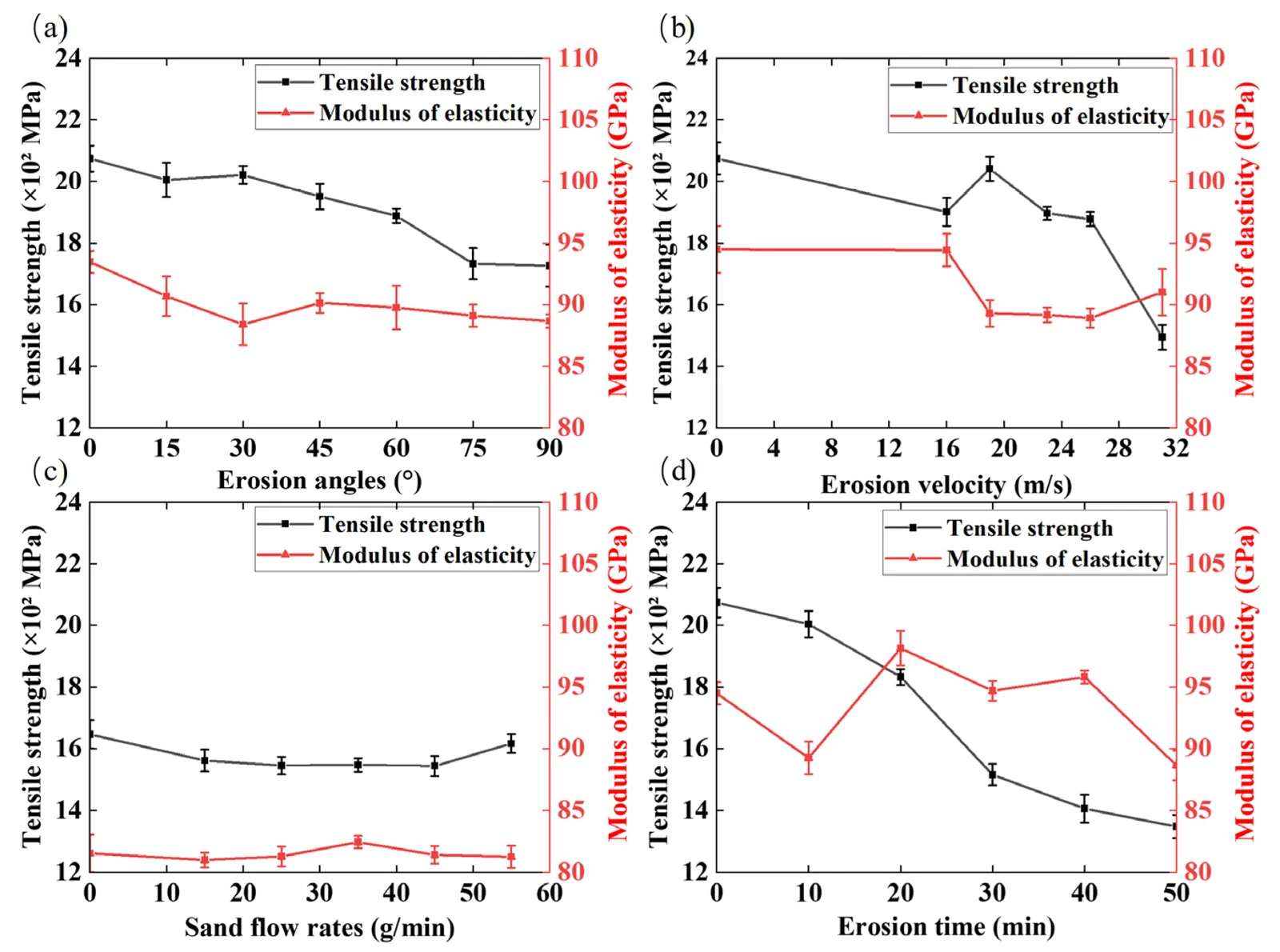
Comparison of residual mechanical properties of GFRP strips under different influencing factors: (**a**) erosion angles, (**b**) erosion velocities, (**c**) sand flow rates, (**d**) erosion times.

In order to study the residual mechanical properties of GFRP strip after aeolian sand erosion, this paper compared and analyzed the tensile strength changes in the material under different erosion angles through direct tensile tests (Figure 5a). All reported strength and modulus values are the mean of three parallel specimens tested for each working condition. The results show that when the erosion angle is less than 45°, the tensile strength only decreases by about 3.3%. When the angle increases to 45° and 60°, the reduction increases to 9%. When it is further increased to 90°, the decline rate of strength is 16.8%, which indicates that the erosion angle has a significant impact on the degradation of tensile properties. Low-angle erosion is mainly caused by friction and cutting, which mainly damages the surface layer of epoxy resin and has low erosion efficiency. At high angles, the direct impact of sand particles leads to brittle peeling of resin, exposure and fracture of fiber, thereby weakening the overall mechanical properties. The elastic modulus changes in GFRP strips under different erosion angles were obtained from extensometer test data. Sand erosion can slightly reduce the elastic modulus, and it continues to decrease with the increase in angle, and the maximum decrease is about 6%, indicating that its influence on elastic modulus is relatively limited, and local damage does not significantly affect the overall stiffness of the material.

Figure 5b illustrates the residual mechanical properties for different erosion velocities. With the increase in speed, the tensile strength showed a downward trend. The strength loss was small when the speed was lower than 26 m/s. When the erosion velocity increased to 26 m/s, the residual tensile strength was approximately 9.5% lower than that of the un-eroded control specimen. At 31 m/s, the strength reduction increased to 28%, and the material properties were significantly degraded. High-speed impact increases the kinetic energy of sand, intensifies the cutting and peeling of epoxy resin, and then threatens the continuity of fiber. The elastic modulus is basically stable under various velocity conditions, and the maximum decrease is only 5.9%, which further confirms that the influence of erosion velocity on the modulus is negligible.

The performance under different sand flow rates is shown in Figure 5c. When the sand flow rate increases within the range of 15 to 45 g/min, the tensile strength decreases gradually by approximately 6%. Beyond 45 g/min, the reduction essentially saturates, with almost no further decrease in tensile strength at 55 g/min, indicating that the degradation of material properties tends to level off once the sand flow rate is sufficiently high. The elastic modulus remained stable under different sand flow rates, indicating that it was minimally affected.

Figure 5d shows the performance changes for varying erosion times. The intensity loss is less than 5% when corroded for 10 min, and the effect is small. At 20 min, the intensity decreased by 11%, and the performance was significantly degraded. At 50 min, the drop was 35%, and the material was severely damaged. Long-time erosion leads to increased cumulative damage, resin peeling and fiber fracture. The elastic modulus decreased by only 6.2% at most after 50 min of erosion, again indicating that the erosion time had no significant effect on the modulus.

In conclusion, wind and sand erosion has a significant impact on the tensile strength of GFRP strips, especially under the action of high angles, high speeds, and long periods of time; the degradation is more obvious. However, its influence on the elastic modulus is relatively small, and local damage does not significantly change the overall stiffness of the material. This apparent contrast between the pronounced strength reduction and the limited modulus change can be explained by the different mechanisms that govern the two properties. Blown-sand erosion is essentially a surface-confined process: the sand particles remove the outer epoxy layer and damage only the near-surface fibers, whereas the majority of the load-bearing fibers through the thickness of the strip remain intact. Because the tensile elastic modulus is dominated by the overall fiber volume fraction acting in parallel, the loss of a thin surface layer reduces the effective load-bearing cross-section only slightly, so the initial stiffness (the slope of the linear stage of the load–displacement response) changes little. Tensile strength, in contrast, is controlled by the most severe local defect: the eroded pits, exposed fiber ends, and stress concentrations at the damaged surface act as crack-initiation sites that trigger premature failure well before the undamaged core reaches its capacity. Consequently, surface erosion can markedly lower the strength while leaving the global modulus almost unchanged, which is consistent with fracture-mechanics-based understanding of notch- and flaw-sensitive brittle composites rather than in conflict with composite micromechanics. A full quantitative separation of these effects would require the complete load–displacement histories of all eroded specimens, which is identified as a direction for further work.

### 3.2. Micromorphological Analysis

**Figure 6:**
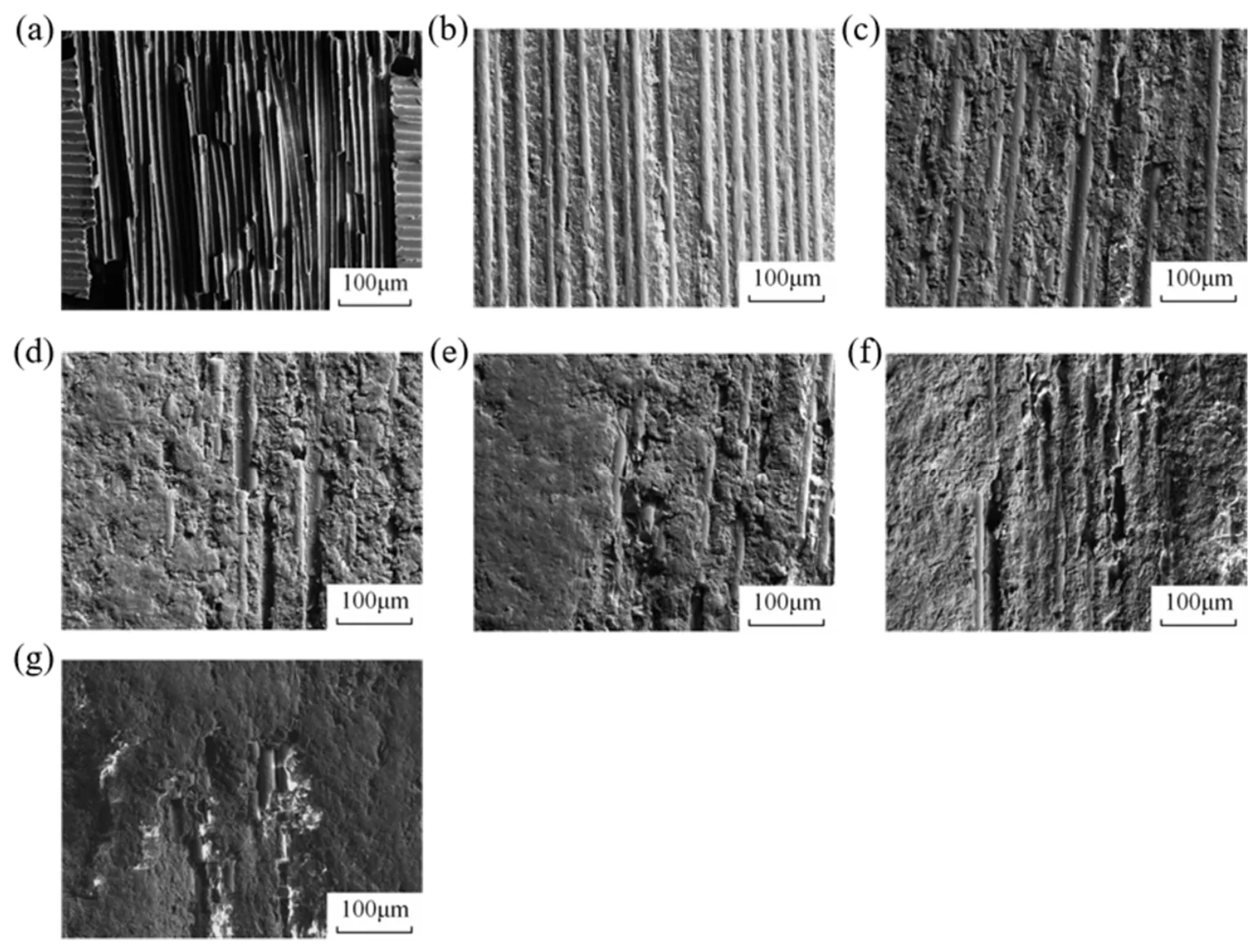
SEM results of GFRP strips under different erosion angles: (**a**) control, (**b**) 15°, (**c**) 30°, (**d**) 45°, (**e**) 60°, (**f**) 75°, (**g**) 90°.

**Figure 7:**
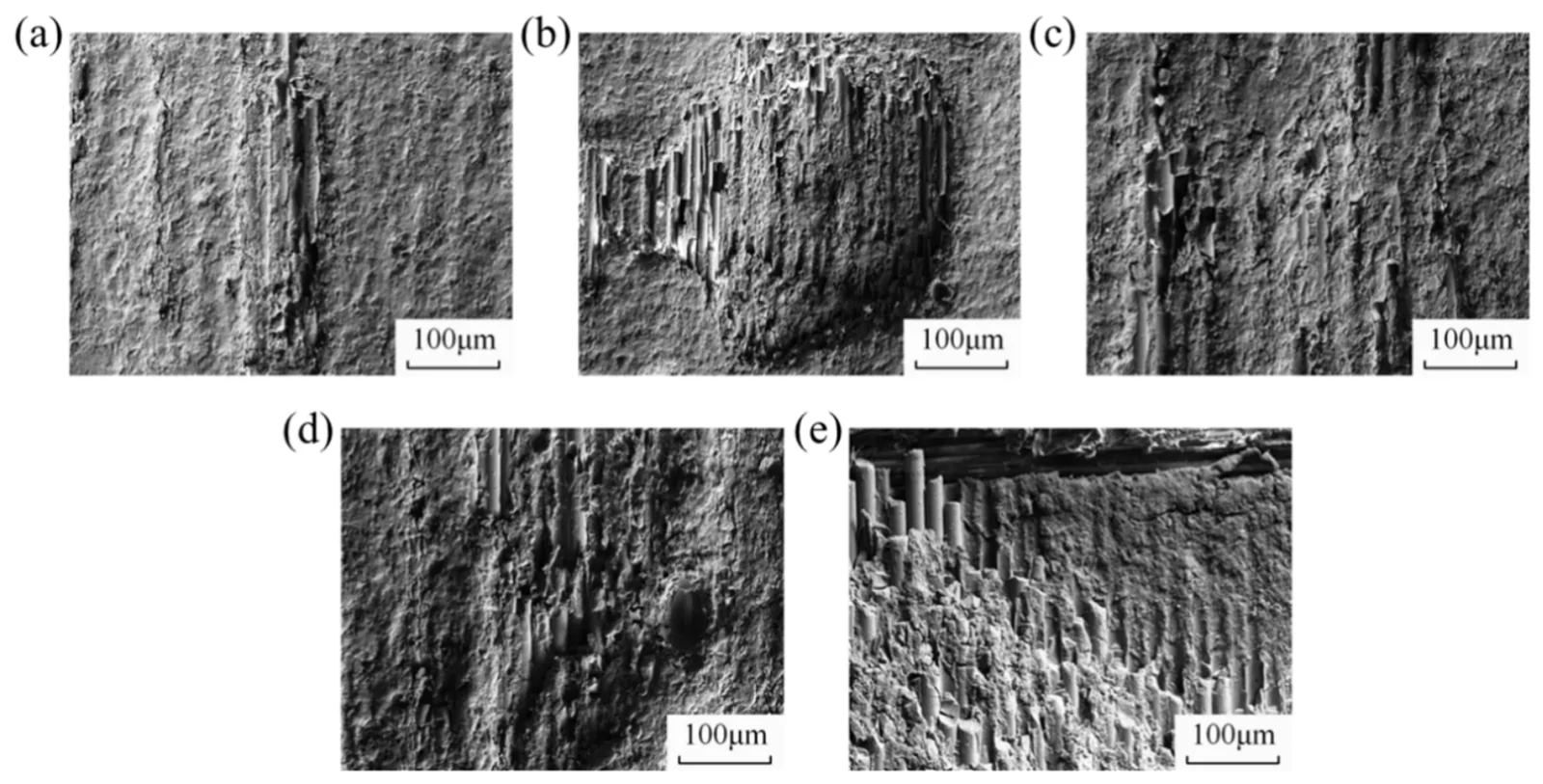
SEM results of GFRP strips under different erosion velocities: (**a**) 16 m/s, (**b**) 19 m/s, (**c**) 23 m/s, (**d**) 26 m/s, (**e**) 31 m/s.

**Figure 8:**
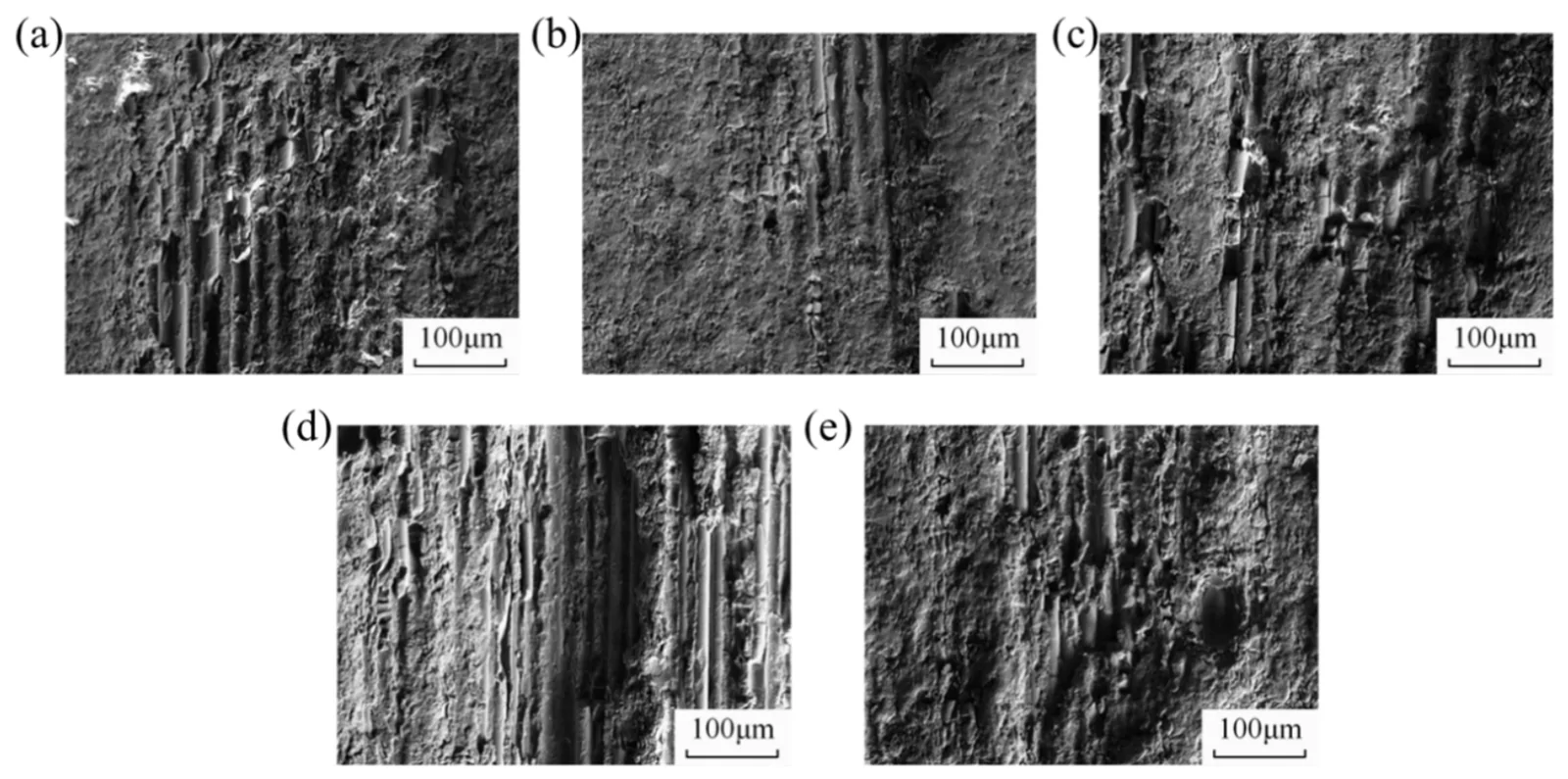
SEM results of GFRP strips under different sand flow rates: (**a**) 15 g/min, (**b**) 25 g/min, (**c**) 35 g/min, (**d**) 45 g/min, (**e**) 55 g/min.

**Figure 9:**
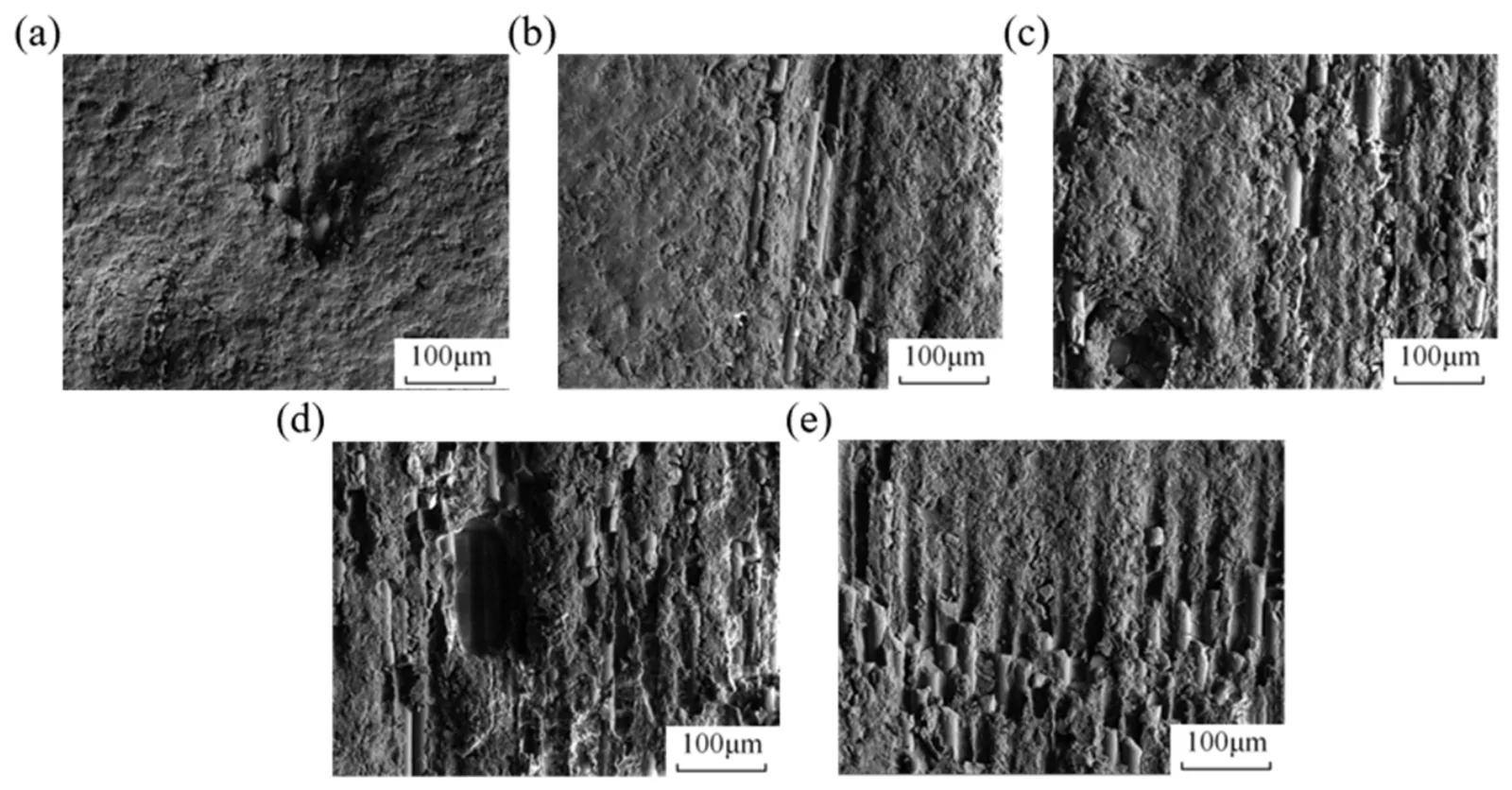
SEM results of GFRP strips under different erosion times: (**a**) 10 min, (**b**) 20 min, (**c**) 30 min, (**d**) 40 min, (**e**) 50 min.

The SEM test results of GFRP strips under different erosion angles were shown in Figure 6. All SEM micrographs shown in Figure 6, Figure 7, Figure 8 and Figure 9 were acquired at a magnification of 1000 times. The GFRP strips used for the test were made of release cloth, so it could be observed from Figure 6a that when the GFRP strips were not subjected to blown-sand erosion, the resin on the surface showed criss-crossed grid-like patterns, which were the patterns of the release cloth. As the erosion angle increases from 15° to 30°, the epoxy adhesive undergoes significant deformation and damage (Figure 6b). However, the texture becomes more defined, and the extent of damage is minimal. When the erosion angle increases from 30° to 45°, and then to 60°, a substantial amount of epoxy adhesive deformation is observed, accompanied by a notable number of adhesive layer failures and the exposure of internal fibers. As the angle increases from 75° to the maximum of 90°, it can be observed that a large number of internal fibers are exposed, and most of them are shattered, resulting in a significant increase in the erosion degree. It is evident that certain specimen areas exhibited gullies resulting from fiber detachment, as well as pits indicative of erosion, as illustrated in Figure 6f,g. In summary, the degree of damage to GFRP strips increases in proportion to the increase in erosion angle, and the effect of erosion angle is significant.

As illustrated in Figure 7, the SEM results of GFRP strips at varying erosion velocities are presented. When the erosion velocity reaches 16 m/s, the epoxy adhesive on the surface of the GFRP strips undergoes minor damage and deformation. With the increase in erosion velocity, especially when the values are 23 m/s and 26 m/s, it can be found that the adhesive layer on the surface of the GFRP strips peeled off and the internal fibers were exposed, and the blown-sand erosion is further intensified. When the erosion velocity reaches 31 m/s, the epoxy adhesive layer of the GFRP strips peels off in a large area, and the internal fibers are exposed and broken in a large number, indicating that the GFRP strips have lost their overall mechanical behavior, as shown in Figure 7e. As the erosion velocity increases, the kinetic energy of the sand particles hitting the GFRP strips increases, which in turn causes a continuous increase in the probability of epoxy adhesion damage and internal fiber fracture damage, and it can be found that the erosion velocity is directly related to the erosion damage of the GFRP strips.

As illustrated in Figure 8, the SEM results of GFRP strips at varying sand flow rates are presented. It has been observed that the adhesive layer on the surface of the GFRP strips peeled off at different sand flow rates, and the internal fibers were exposed. The damage to the internal fibers increased with the sand flow rates, especially at rates of 45 g/min and 55 g/min. The epoxy adhesive layer of the GFRP strips exhibited significant delamination, exposing the internal fibers and leading to substantial fiber fracture. This outcome suggests that the overall stress performance of the GFRP strips was compromised, as evidenced by the observations presented in Figure 8d,e. The increase in sand flow rates has been shown to result in an elevated probability of sand particles impacting GFRP strips. This, in turn, has been demonstrated to cause epoxy adhesive damage and internal fiber fracture. Therefore, it can be concluded that the sand flow rate has a significant effect on the results of erosion damage to GFRP strips.

The SEM results of GFRP strips under different erosion times are presented in Figure 9. At relatively short erosion times of 10 min and 20 min, only minor deformation and limited peeling of the surface epoxy adhesive layer are observed, and the internal fibers remain largely protected. As the erosion time increases to 30, 40 and 50 min, the cumulative impact of sand particles results in progressively more extensive peeling of the epoxy adhesive layer, accompanied by exposure and fracture of the internal fibers; the most pronounced damage occurs at 50 min, as shown in Figure 9e. These observations indicate that prolonged erosion time aggravates the accumulation of surface and fiber damage, which is consistent with the continuous reduction in tensile strength with erosion time reported in Section 3.1. Taken together, the SEM observations and the residual-strength measurements exhibit a consistent, monotonic correspondence between the severity of microscopic damage and the macroscopic strength loss. When the damage remains confined to deformation and limited peeling of the surface epoxy layer (low erosion angle, low velocity, or short erosion time), the tensile strength decreases only slightly (about 3.3% below 45°, less than 5% at 10 min). As the damage progresses to extensive adhesive layer peeling and partial exposure of the internal fibers (intermediate conditions), the strength loss increases to roughly 9–11%. When the SEM images show large-area resin removal together with widespread fiber exposure and fracture (90° erosion angle, 31 m/s, or 50 min erosion), the strength loss reaches its maximum of 16.8%, 28% and 35%, respectively. This ordered correspondence shows that the SEM morphology is not merely an isolated qualitative illustration but tracks the same damage-accumulation process that governs the measured strength degradation; a strictly quantitative correlation (e.g., fiber-break density or eroded area versus strength retention) would require automated image quantification of the micrographs and is left for future work.

### 3.3. Strain Analysis in GFRP Strips

According to the photographic evidence gathered through the DIC technique during the test, the strain field cloud of the GFRP strips following blown-sand erosion at varying angles can be calculated and generated for each load level, as illustrated in Table 3 (with the erosion velocity fixed at 26 m/s, the sand flow rate at 55 g/min, and the erosion time at 30 min). Here, P_u_ (denoted as P_u_ in the strain-distribution analysis below) represents the ultimate tensile load of the GFRP specimen, and 0.2P_u_ to P_u_ denote the corresponding load levels. It has been demonstrated that, in circumstances where the erosion angle is less than 45°, the deformation of the GFRP strips within the erosion area exhibits greater uniformity. There is an absence of discernible stress concentration phenomena. It can be hypothesized that when the angle of blown-sand erosion is minimal, the damage caused by blown-sand erosion on GFRP strips is negligible, and the overall deformation of the specimen is more uniform. As the erosion angle increases, particularly at 75° and 90°, it becomes evident that the deformation of the material within the erosion area is non-uniform. This is particularly pronounced at higher load levels, where the deformation in the damaged area is accentuated. Upon reaching its peak load, each specimen exhibited a conspicuous stress concentration phenomenon at the core of the erosion area, suggesting that the damage in this region has accumulated significantly. Consequently, the first material damage is observed, which subsequently affects the overall mechanical behavior of the specimen. It is evident that as the erosion angle increases, the stress concentration on the surface of the GFRP strips becomes more obvious, resulting in a subsequent decrease in the material’s tensile strength due to localized deterioration of its properties.

The results of the DIC calculations are shown in Table 4 (with the erosion angle fixed at 90°, the sand flow rate at 55 g/min, and the erosion time at 30 min). When the erosion velocity is 19 m/s, the strain of GFRP strips is uniformly distributed, and the deformation of each area of the material is relatively similar even when the load reaches the peak, which indicates that the damage of the material is not obvious at the lower erosion velocity. When the erosion velocity approaches 23 m/s or 26 m/s, the deformation in the erosion center region (i.e., the central area of the eroded zone directly facing the sand jet, where damage accumulation is most concentrated) is negligible at lower stress levels. As the stress level increases, the deformation in the damage region becomes marked. Ultimately, the damage is confined to the erosion center region. It has been demonstrated that the erosion process will exert a direct influence on the remaining mechanical properties of GFRP strips. At velocities of 31 m/s, it is evident that the deformation in the center of the erosion area is consistently more obvious from the lower load level to the peak value. The erosion effect of high-speed airflow containing sand has been shown to directly impact the damage evolution and progression of GFRP strips. It is evident that as the erosion velocity increases, the stress concentration phenomenon on the surface of the GFRP strips becomes more obvious, resulting in the localized deterioration of the material’s properties and a consequent decrease in tensile strength.

Based on the DIC technique, the strain cloud of the GFRP strips after blown-sand erosion with different sand flow rates can be calculated and generated under various loading levels, as shown in Table 5 (with the erosion angle fixed at 90°, the erosion velocity at 26 m/s, and the erosion time at 30 min). At a sand flow rate of 15 g/min, the strain of the GFRP strips exhibited uniform distribution, and the deformation of each material area is relatively similar, even at peak load, which suggests that the sand flow rate does not result in substantial damage to the material. As the sand flow rate continues to increase, the deformation in the erosion center region is not significant at relatively low loading levels. The deformation began to be prominent when the loading level increased. Moreover, the final damage all occurred in the erosion center region. It has been demonstrated that an increase in the sand flow rate results in a discernible enhancement of the stress concentration phenomenon on the surface of the GFRP strips. It could be concluded that a localized deterioration of the material’s properties would cause the decline of the material’s tensile strength.

The DIC results are shown in Table 6 (with the erosion angle fixed at 90°, the erosion velocity at 26 m/s, and the sand flow rate at 55 g/min). Under varying conditions of erosion time, the deformation in the erosion center region is negligible at low loading levels. When the load level increases, the deformation in the damage region becomes pronounced, and the final damage occurs in the erosion center region. It has been confirmed that the erosion effect will directly impact the residual mechanical properties of GFRP strips. As the erosion time reaches 50 min, it becomes evident that the deformation in the center of the erosion region is consistently more pronounced. The erosion effect of high-speed airflow with sand directly influences the damage evolution and damage process of the GFRP strips. It can be seen that with the increase in the erosion time, the stress concentration phenomenon on the surface of the GFRP strips becomes more and more obvious, and the localized deterioration of the properties leads to the continuous decrease in the tensile strength of the GFRP strips.

### 3.4. Strain Distribution in the Erosion Area

**Figure 10:**
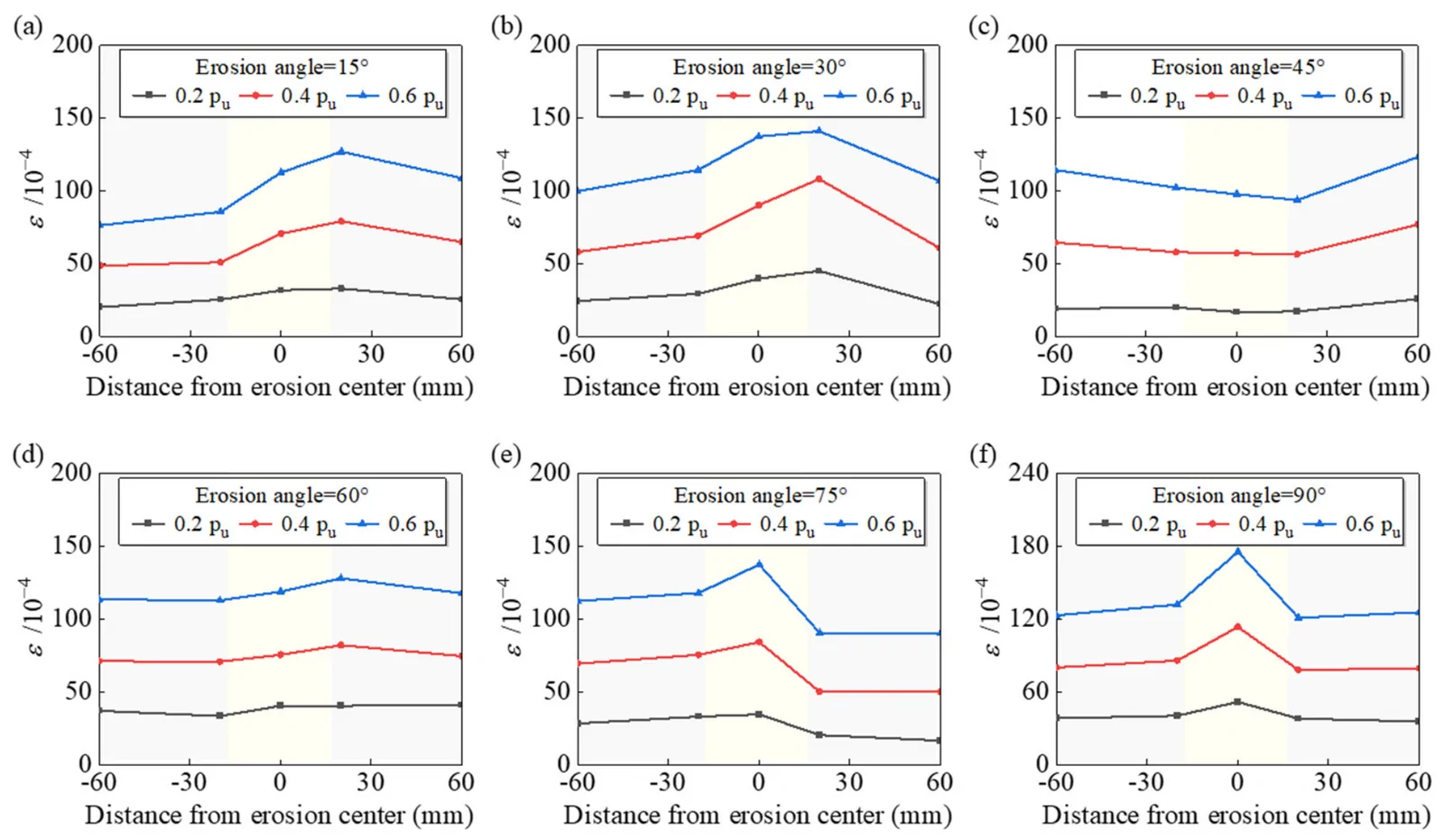
Strain distribution of GFRP strips under different erosion angles: (**a**) 15°, (**b**) 30°, (**c**) 45°, (**d**) 60°, (**e**) 75°, (**f**) 90°.

A study of the test data of strain gauges in the blown-sand erosion area reveals the strain distribution curves of GFRP strips in the damaged area under various loading levels and different erosion angles. These curves are illustrated in Figure 10.

When the load level is minimal, specifically at the 0.2P_u_ level, it becomes evident that the strain data at varying measurement points exhibit greater proximity to each other. As the loading level increases, reaching 0.4P_u_, an observed increase in strain data near the center of the erosion region suggests potential damage deformation of the material. As the load level reaches 0.6P_u_, the strain values in the erosion center region exhibit a marked increase compared to other regions, suggesting that the damage deformation of the material in this region persists and accumulates. A comparison of the distribution graphs of the curves reveals that as the erosion angle increases, the strain distribution law becomes more pronounced. This observation indicates that as the erosion angle increases, the damage and deformation of the erosion center region also increase in severity.

**Figure 11:**
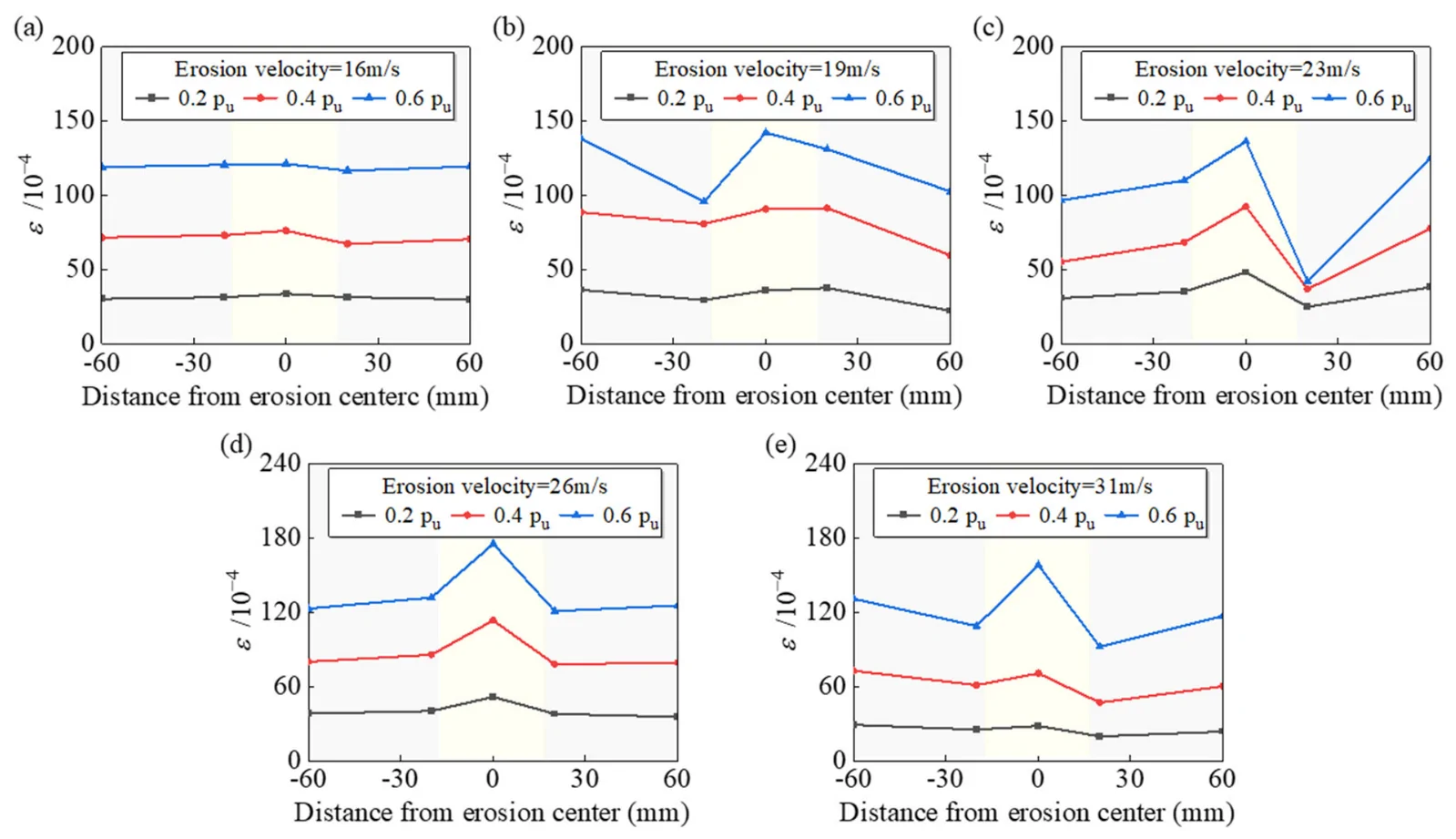
Strain distribution of GFRP strips under different erosion velocities: (**a**) 16 m/s, (**b**) 19 m/s, (**c**) 23 m/s, (**d**) 26 m/s, (**e**) 31 m/s.

The strain distribution curves of GFRP strips with different erosion velocities are plotted for each load level in the damage area as shown in Figure 11.

When the erosion velocity was 16 m/s, the strains of the GFRP strips were basically similar in different areas even under different load levels, indicating that lower erosion velocities could not cause significant damage. With the increase in the erosion velocity to 23 m/s or 26 m/s, it can be observed that the strain value near the center of the erosion area is larger, indicating that there is more deformation and damage in the initial damage area during the process of axial tension. It should be noted that a local increase in strain is observed at the measurement point located approximately +60 mm from the erosion center in Figure 11c. This non-monotonic feature indicates that the strain distribution is not perfectly symmetric about the nominal erosion center and may be associated with the spatially heterogeneous distribution of erosion-induced resin and fiber damage. In addition, because the strain gauges provide measurements only at discrete locations, this isolated increase is not interpreted as evidence of a systematic secondary stress concentration zone. Observing Figure 11e, the deformation of each part of the GFRP strips is closer when the load level is 0.2P_u_. As the load level increases to 0.4P_u_, it becomes evident that the deformation in the center of the erosion area increases significantly, with a substantially higher value when compared to the other adjacent locations. When the load level increases to 0.6P_u_, the deformation in the initial damage region is more obvious, significantly larger than the corresponding values at other locations, and the stress concentration phenomenon emerges. Comparing the distribution of the curves horizontally, it can be deduced that with the increasing of the erosion velocity, the deformation of the erosion center region is more and more prominent compared with the other adjacent regions, which indicates that the erosion velocity will adversely affect the residual mechanical properties of the GFRP strips.

**Figure 12:**
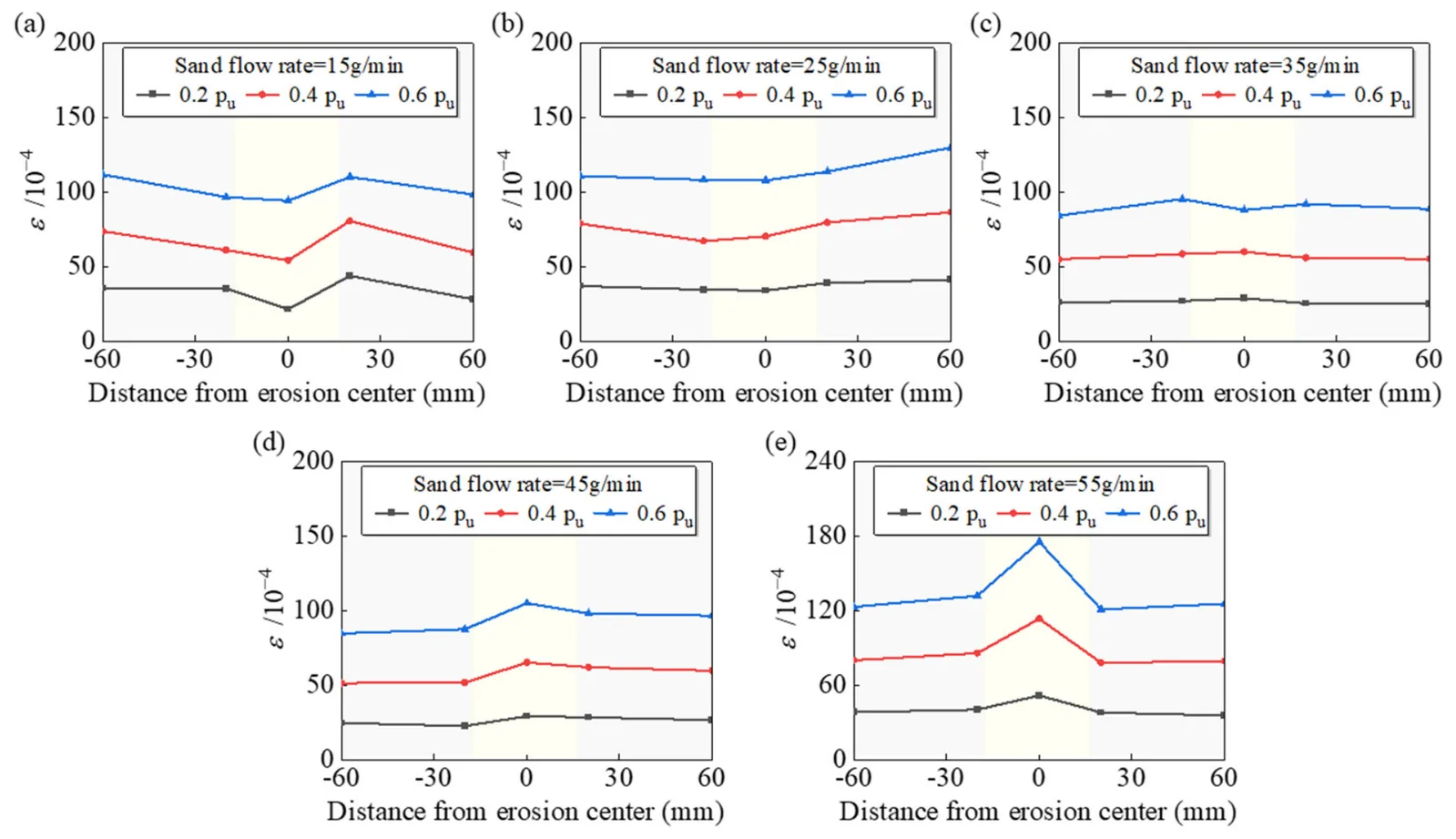
Strain distribution of GFRP strips under different sand flow rates: (**a**) 15 g/min, (**b**) 25 g/min, (**c**) 35 g/min, (**d**) 45 g/min, (**e**) 55 g/min.

In Figure 12, the strain distribution curves in the damage region are plotted for different sand flow rate conditions of GFRP strips under various load levels. When the sand flow rate is less than 35 g/min, the strains of the GFRP strips are essentially analogous in different areas, even under varying load levels. This finding suggests that lower sand flow rates do not result in substantial damage to the material. As the sand flow rate increases to 45 g/min or 55 g/min, it can be observed that the strain values near the center of the erosion region are larger, and the corresponding values increase significantly as the load continues to increase, which indicates that more serious deformation and damage occur in the initial damage region under direct tension. A horizontal comparison of the distribution curves reveals that the deformation in the center of the erosion region becomes increasingly obvious with an increase in the sand flow rate, suggesting deleterious effects of the sand flow rate on the residual mechanical properties of the GFRP strips.

**Figure 13:**
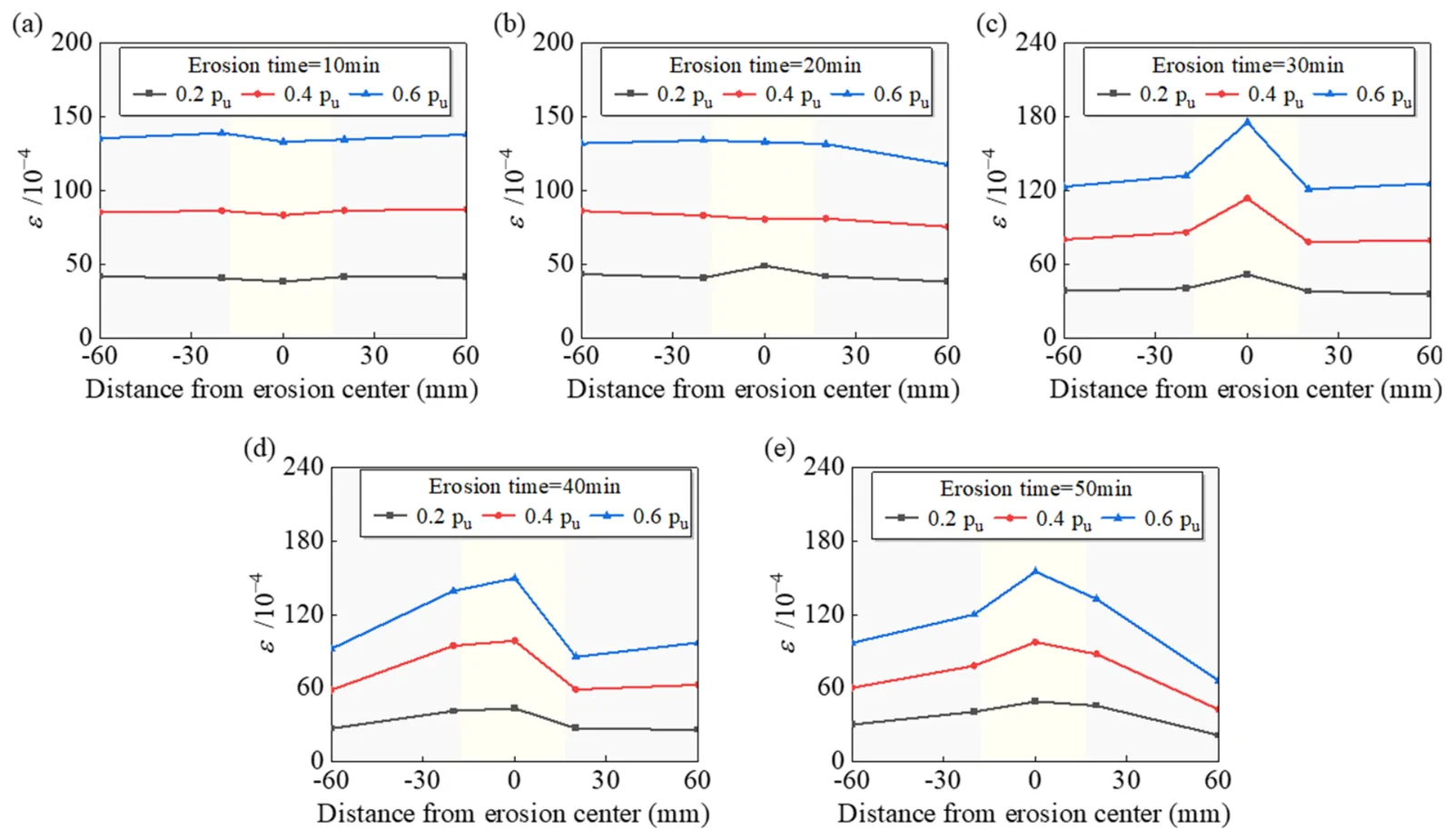
Strain distribution of GFRP strips under different erosion times: (**a**) 10 min, (**b**) 20 min, (**c**) 30 min, (**d**) 40 min, (**e**) 50 min.

The strain distribution curves of the material in the damage region for different erosion times of GFRP strips under different loading levels are plotted in Figure 13. When the erosion time was set at 10 or 20 min, the strains are almost the same across the GFRP strip regions. Though the loading level goes up, there is no obvious difference. As the erosion time reaches 30 to 50 min, it can be observed that the strain value near the center of the erosion region is larger, indicating that the initial damage region undergoes more serious deformation and damage. As illustrated in Figure 13e, the deformation of each part of the GFRP strips is more closely aligned when the load level is 0.2P_u_. As the load level increases to 0.4P_u_, it is evident that the deformation in the center of the erosion area increases significantly, with a substantially higher value when compared to other adjacent locations. When the load level reaches 0.6P_u_, the deformation in the initial damage region is marked, significantly larger than the corresponding values at other locations, and the stress concentration phenomenon emerges. A horizontal comparison of the distribution graphs of the curves reveals that as the erosion time increases, the deformation of the erosion center region becomes more pronounced compared to the other adjacent regions. This observation suggests that the erosion time will have a substantial negative impact on the residual mechanical properties of the GFRP strips.

## 4. The Post-Erosion Residual-Property Evaluation

### 4.1. The Prediction Model Establishment

**Figure 14:**
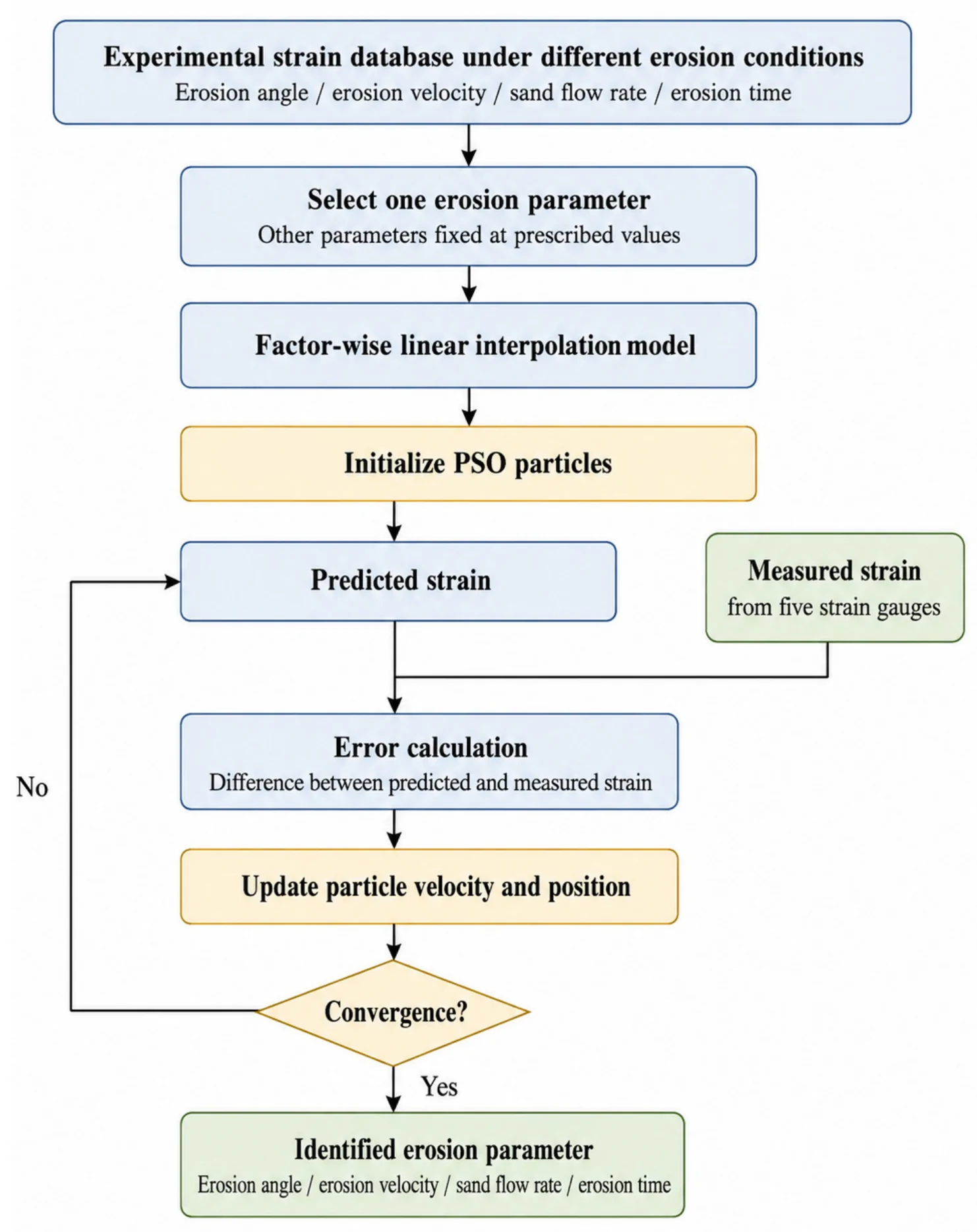
Factor-wise inverse identification model of blown-sand erosion parameters based on the particle swarm optimization (PSO) algorithm.

Erosion angle, erosion velocities, sand flow rate, and erosion time are critical factors influencing the initiation and evolution of material damage. Characterizing the erosion behavior can reveal the full progression of material degradation from initial damage to severe failure, thereby providing a basis for service life prediction of materials. The prediction model consists of a factor-wise linear interpolation model for predicting the strain response and a PSO-based inverse model for identifying the corresponding erosion parameter, as shown in Figure 14. For each identification process, one erosion parameter is selected, while the remaining parameters are fixed at their prescribed values.

Based on the experimental design, separate interpolation relationships were established between each erosion factor and the material strain response, while the remaining three erosion factors were fixed at their prescribed values, as follows:

(1) $$\left\{\begin{array}{c} \varepsilon_{i}=f_{\theta ,i}(\theta ),\;v_{e}=26\;m/s,\;v_{s}=55\;g/\min,\;t=30\;\min\\ \varepsilon_{i}=f_{v_{e},i}(v_{e}),\;\theta =90{}^{\circ},\;v_{s}=55\;g/\min,\;t=30\;\min\\ \varepsilon_{i}=f_{v_{s},i}(v_{s}),\;\theta =90{}^{\circ},\;v_{s}=26\;g/\min,\;t=30\;\min\\ \varepsilon_{i}=f_{t,i}(t),\;\theta =90{}^{\circ},\;v_{e}=26\;g/\min,\;v_{s}=55\;g/\min \end{array}\right.$$

where *θ*, *v*_e_, *v*_s_, and *t* denote the erosion angle, erosion velocity, sand flow rate, and erosion time, respectively; *f_θ,i_*, *f_v_*_e,*i*_, *f_v_*_s,*i*_, and *f_t_*_,*i*_ denote the corresponding factor-wise interpolation functions; and *ε_i_* is the strain measured by strain gauge *i* (*i* = 1, 2, 3, 4, 5).

In solving the inverse problem of erosion characteristics, the particle swarm optimization (PSO) intelligent optimization algorithm is used to inversely identify the blown-sand erosion parameters through the tested GFRP strain. This method regards the erosion behavior as a particle in the particle swarm and continuously adjusts the parameter value through an iterative optimization process. First, the particle swarm is initialized, and the population position and particle velocity are randomly generated. Then, the error between the predicted strain and the tested strain corresponding to each erosion characteristic is calculated as the fitness function, that is

(2) $$\mathrm{Fitness}\left(\xi \right)=\sqrt{\sum_{i=1}^{5}{\left[\varepsilon_{i}^{\mathrm{pred}}\left(\xi \right)-\varepsilon_{i}^{\mathrm{test}}\right]}^{2}}$$

where *ξ* denotes the erosion parameter to be identified in the current inverse-analysis run, i.e., *θ*, *v*_e_, *v*_s_, or *t*; *ε_i_*^pred^(*ξ*) and *ε_i_*^test^ denote the predicted and measured strains at strain gauge *i*, respectively. For each run, only one erosion parameter is optimized, while the remaining three parameters are fixed at their prescribed values.

During each iteration, the velocity and position of each particle are updated to optimize the erosion behavior parameters, as governed by the equations shown in Figure 14. Specifically, the search direction and step size of the *j*-th particle are dynamically adjusted based on its personal best solution *p_j_* and the global best solution of the swarm *gb*. The update process is defined as

(3) $$v_{j}\left(k+1\right)=\omega v_{j}\left(k\right)+c_{1}r_{1}\left({pb}_{j}-x_{j}\left(k\right)\right)+c_{2}r_{2}\left(gb-x_{j}\left(k\right)\right)$$

(4) $$x_{j}\left(k+1\right)=x_{j}\left(k\right)+v_{j}\left(k\right)$$

where $x_{j}$ and $v_{j}$ denote the position and velocity of the *j*-th particle; *t* is the iteration index; $c_{1}$ and $c_{2}$ are the cognitive and social learning factors; and $r_{1},r_{2}\in \left[0,1\right]$ are random variables. To ensure search stability, the particle velocity is constrained within the range $v_{j}\in \left[-v_{\max},v_{\max}\right]$, with $v_{\max}$ set to 1.0 to prevent excessive oscillation.

The PSO hyperparameters were determined through a preliminary sensitivity analysis, consistent with the established literature. For each factor-wise one-parameter inverse identification problem, a population size of 10 and a maximum iteration count ($K_{max}$) of 40 were found sufficient for the fitness function to converge below the preset threshold, further increases in these values yielded no meaningful reduction in identification error while significantly increasing computational costs. The learning factors were set to $c_{1}$ = 2.5 and $c_{2}$ = 3.0, prioritizing global information to accelerate convergence in this low-dimensional space. The inertia weight ω follows a nonlinear decay strategy to ensure a smooth transition from global exploration in early iterations to local exploitation in later stages, as defined by

(5) ω=ωmin(ωmaxωmin)1−(k/Kmax)2

where $\omega_{max}$ = 0.9 and $\omega_{min}$= 0.4, and *k* represents the current iteration count. Since the PSO search is inherently stochastic and sensitive to the initial particle swarm, each inverse identification was repeated 20 times. The mean value of these 20 runs is reported to quantify the influence of algorithmic randomness on the identified parameters. The variability among these repeated runs reflects only the stochastic nature of the PSO algorithm and does not represent specimen-to-specimen experimental variability or measurement uncertainty.

### 4.2. The Prediction Results of the GFRP Strain

**Figure 15:**
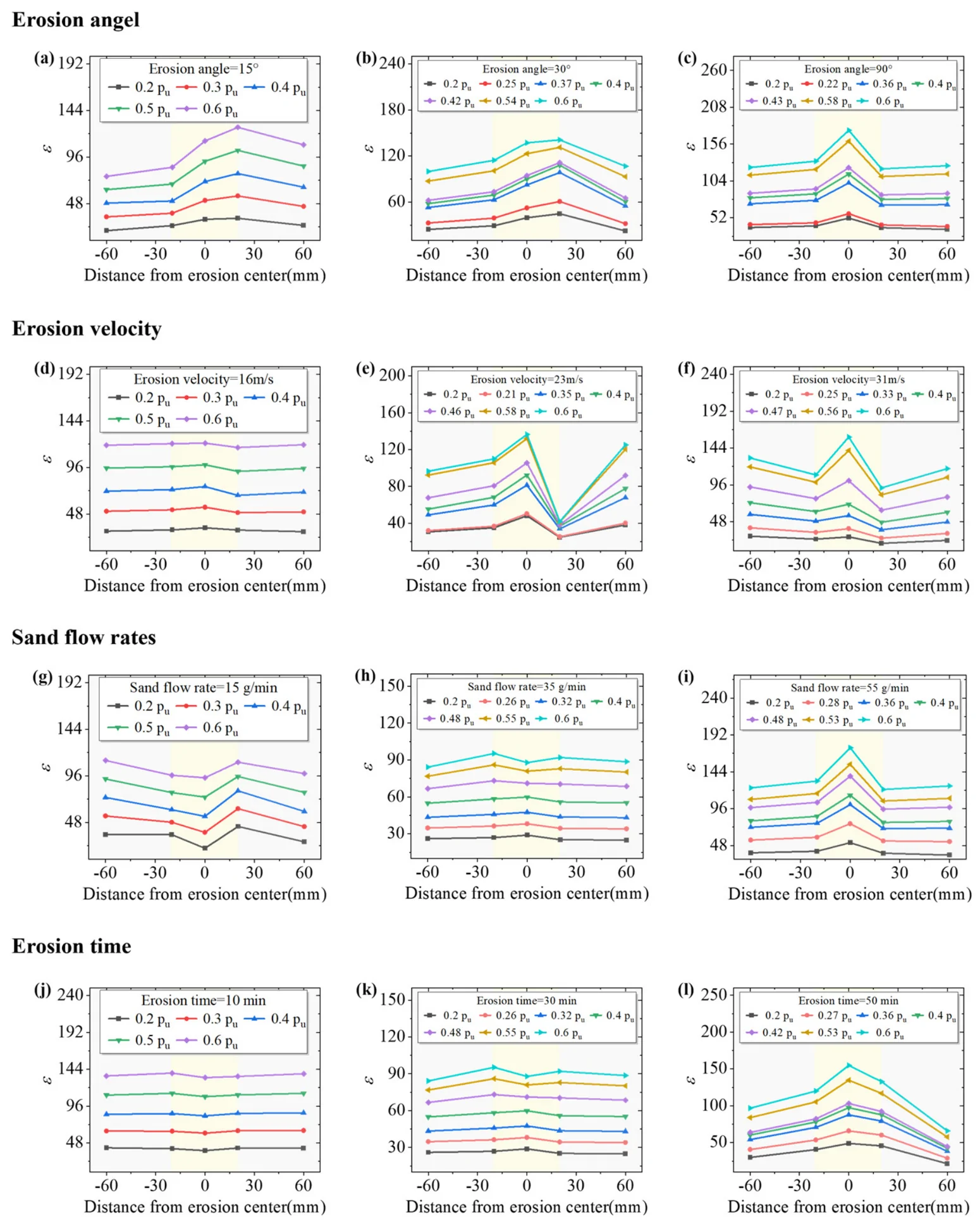
GFRP strain prediction results under different erosion conditions. Erosion angle: (**a**) 15°, (**b**) 30°, (**c**) 90°. Erosion velocity: (**d**) 16 m/s, (**e**) 23 m/s, (**f**) 31 m/s. Sand flow rate: (**g**) 15 g/min, (**h**) 35 g/min, (**i**) 55 g/min. Erosion time: (**j**) 10 min, (**k**) 30 min, (**l**) 50 min.

Based on the factor-wise interpolation relationships established in Section 4.1, the strain responses of the GFRP strips under the investigated erosion conditions are predicted. Figure 15 shows the predicted strain responses under different erosion conditions.

Figure 15a shows the prediction results of the GFRP material strain under different erosion angles, where the erosion time is 30 min, the sand flow rate is 55 g/min, and the erosion velocity is 26 m/s. When the erosion angles are 15°, 30°, and 90°, the predicted values of 0.2Pᵤ, 0.4Pᵤ, and 0.6Pᵤ are consistent with the tested strains shown in Figure 10, while the predicted strain values at any point under the intermediate load show a smooth transition trend that conforms to the law of material damage evolution. Figure 15d–f shows the strain prediction results of GFRP materials under different erosion velocities, with the erosion angle fixed at 90°, the sand flow rate at 55 g/min, and the erosion time at 30 min. For erosion velocities of 16 m/s, 23 m/s, and 31 m/s, the predicted strain values at 0.2Pᵤ, 0.4Pᵤ, and 0.6Pᵤ are consistent with the experimental strain data shown in Figure 11.

Under the condition of erosion velocity of 16 m/s, the predicted strain increments at the 0.3*Pᵤ* and 0.5*Pᵤ* load points are uniform. When the erosion velocity is 23 m/s and 31 m/s, the predicted strain at any load point also shows a reasonable jump. Figure 15g–i presents the strain prediction results under different sand flow rates, with the erosion angle fixed at 90°, the erosion velocity at 26 m/s, and the erosion time at 30 min. Figure 15j–l presents the corresponding results under different erosion times, with the erosion angle fixed at 90°, the erosion velocity at 26 m/s, and the sand flow rate at 55 g/min. For the investigated sand flow rates and erosion times, the predicted strain values at 0.2Pᵤ, 0.4Pᵤ, and 0.6Pᵤ are consistent with the experimental strain data shown in Figure 12 and Figure 13, respectively, while the interpolated strain responses at the intermediate load levels exhibit continuous trends. These results indicate that the interpolation model can reproduce the strain-response trends of the calibration data. Its generalization performance for unseen conditions is evaluated separately in Section 4.4.

### 4.3. In-Sample Inverse Identification Results

**Figure 16:**
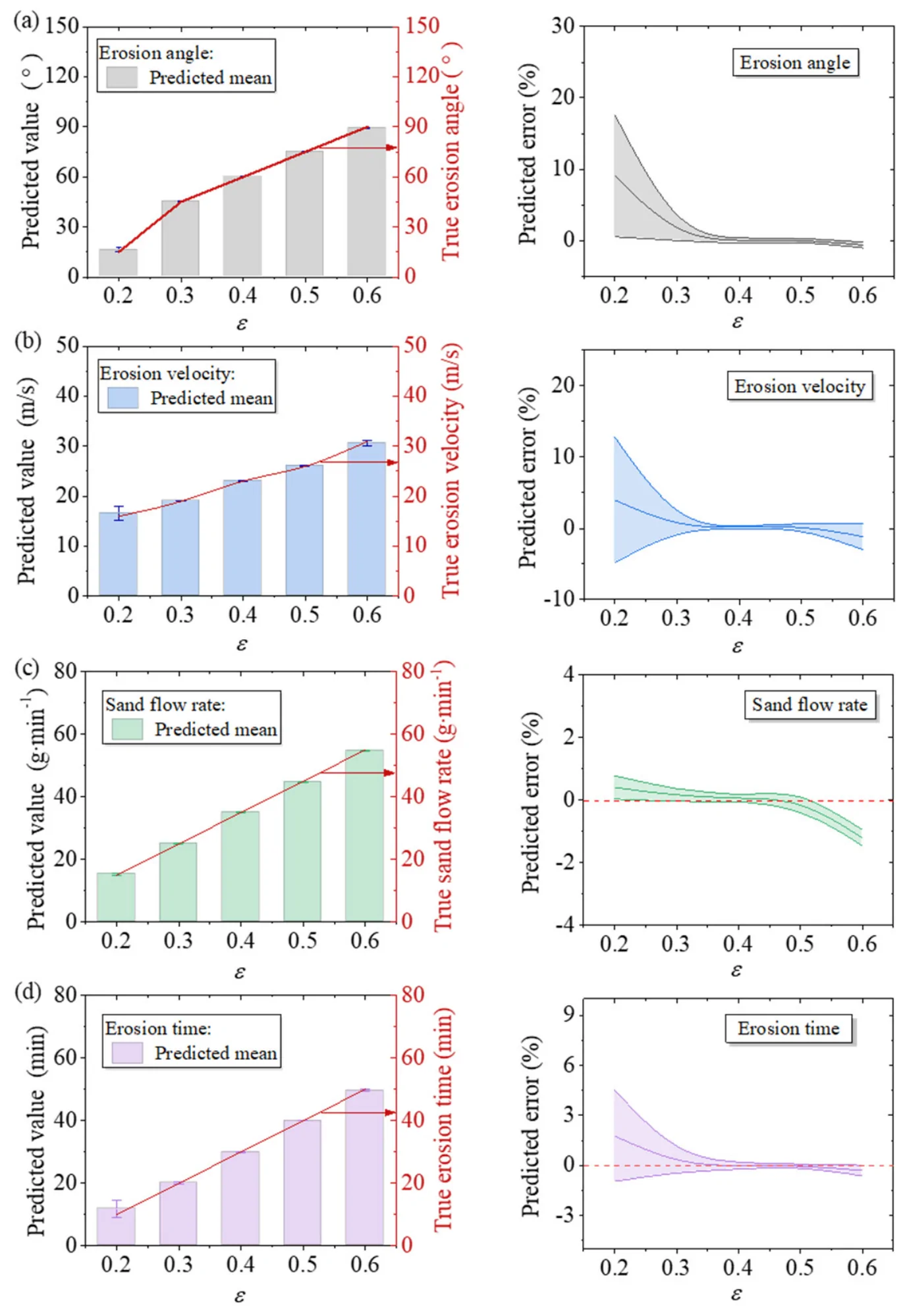
The in-sample inverse identification results of blown-sand erosion behavior: (**a**) erosion angle, (**b**) erosion velocity, (**c**) sand flow rate, (**d**) erosion time.

The inverse identification model of blown-sand erosion parameters based on the PSO algorithm is used to predict the blown-sand erosion behavior. In order to overcome the randomness of the PSO algorithm, multiple predictions are carried out based on the tested strain values in the evaluation method, and here 20 predictions are carried out. Figure 16 shows the prediction results of the blown-sand erosion behavior based on the tested GFRP strain values. In the following, the “true” erosion angle, velocity, sand flow rate, and time refer to the actual values set in the erosion experiments (i.e., the reference experimental values), against which the inversely identified values are compared.

Figure 16a shows the prediction results of the erosion angle, where the erosion time is 30 min, the sand flow rate is 55 g/min, and the erosion velocity is 26 m/s. As can be seen from Figure 16a, when the tested strain is 0.2P_u_, there is a deviation between the mean of the predicted erosion angle and the true erosion angle, and the relative error between the predicted erosion angle and the true erosion angle is in the range of 0–18%. As the tested strain value increases, the relative error of the predicted erosion angle gradually decreases.

Figure 16b shows the prediction results of erosion velocity, where erosion time is 30 min, the sand flow rate is 55 g/min, and the erosion angle is 90°. As can be seen from Figure 16b, when the tested strain is 0.2P_u_, there is a deviation between the mean of the predicted erosion velocity and the true erosion velocity, and the relative error between the predicted erosion velocity and the true erosion velocity is in the range of −5–13%. The relative error of the predicted erosion velocity gradually decreases with the increase in the tested strain value.

Figure 16c shows the predicted results of the sand flow rates, where the erosion time is 30 min, the erosion velocity is 26 m/s, and the erosion angle is 90°. It can be seen from Figure 16c that when the tested strain is in the range of 0.2Pᵤ–0.6Pᵤ, the mean of the predicted sand flow rates is almost consistent with the true sand flow rates, and the relative error between the predicted sand flow rates and the true sand flow rates is within 5%.

Figure 16d shows the prediction result of erosion time, where the sand flow rate is 55 g/min, the erosion velocity is 26 m/s, and the erosion angle is 90°. It can be seen from Figure 16d that when the tested strain is in the range of 0.2P_u_–0.6 P_u_, the mean of the predicted erosion time is almost consistent with the true erosion time, and the relative error between the identified erosion time and the true erosion time is within 10%; it should be noted that this value is obtained in-sample, whereas the independent cross-validation in Section 4.4 (Table 7) gives substantially larger errors for unseen conditions.

The present results demonstrate the feasibility of using strain responses for factor-wise inverse identification of blown-sand erosion parameters under the investigated laboratory conditions. It should be emphasized that P_u_ in the present study represents the specimen-specific ultimate tensile load obtained from the destructive tensile test, and the strain responses at 0.2P_u_–0.6P_u_ are therefore normalized using a quantity that is known only after failure testing. Consequently, the present P_u_-normalized inverse identification procedure should be regarded as a laboratory proof of concept and cannot be directly applied to an in-service GFRP member whose ultimate load is unknown. For future engineering applications, the method could be reformulated using a known non-destructive reference load P_ref_, such as a prescribed proof or service load, or by directly using absolute load–strain pairs as the model input. Such a field-oriented formulation would require an expanded experimental database and independent validation before practical application. In addition, because the present experiments were conducted using a one-factor-at-a-time design, the inverse identification is performed for one erosion factor at a time while the remaining factors are maintained at their prescribed baseline values; simultaneous identification of multiple independently varying erosion factors has not been validated in this study.

### 4.4. Cross-Validation and Applicable Range of the Inverse Model

It should be emphasized that the identification results in Figure 16 are obtained for conditions that are themselves part of the strain database used to build the forward mapping. In this in-sample sense, they mainly reflect the ability of the model to reproduce the data it was constructed from. To assess the genuine generalization ability of the inverse model, a leave-one-condition-out cross-validation was additionally performed: for each erosion factor, one interior parameter level was removed from the database in turn, the forward strain-parameter mapping was rebuilt from the remaining levels, and the measured strain field of the removed level was then used as the input to recover its parameter value; each identification was repeated 20 times to average out the randomness of the PSO search. The results are summarized in Table 7. Within the tested grid (in-sample), the mean relative error of the identified parameters is only about 4.2%, consistent with the good agreement shown in Figure 16. For genuinely unseen conditions, however, the mean relative error rises to about 31.8% and is largest for the erosion angle and the sand flow rate. The factor-specific cross-validation errors indicate different levels of parameter identifiability. The larger errors for erosion angle and sand flow rate suggest that non-monotonic or weak strain–parameter relationships may lead to similar strain responses for different parameter values, thereby reducing the uniqueness of the inverse solution. The low in-sample error mainly reflects reconstruction of the calibration data and should not be interpreted as predictive accuracy for new conditions. The leave-one-condition-out results indicate limited generalization to unseen single-factor conditions even when the held-out parameter level lies within the nominal experimental bounds. Because the database was generated using an OFAT design, conditions involving two or more simultaneously off-baseline factors have not been experimentally validated and cannot be regarded as interpolation points of the present model. Enlarging the experimental database with additional parameter levels and an independent test set is required before the method can be used as a quantitative predictor for arbitrary erosion conditions. The reported results are based on the mean values of three parallel specimens; however, specimen-to-specimen variability was not quantitatively evaluated and should be incorporated into future uncertainty analyses.

## 5. Conclusions

Erosion angle, erosion velocities, sand flow rate, and erosion time are critical factors influencing the initiation and evolution of material damage. This paper conducted blown-sand erosion experiments on the GFRP strips under different erosion environments and analyzed the residual mechanical properties of the GFRP strips under different erosion environments. In addition, this paper established a strain-data-driven, interpolation-based inverse identification model to recover the blown-sand erosion parameters of GFRP strips from the measured strain field within the tested range. The conclusions of this paper are as follows.

(1) With the increase in erosion angle, the epoxy colloid layer on the surface of GFRP strips exfoliated intensely, and the internal fibers were gradually exposed and fractured. Increasing the erosion velocity or time significantly enhances the probability of epoxy colloid damage and internal fiber fracture, while increasing the sanding rate mainly aggravates the damage of internal fibers. SEM results clearly present the differences in damage morphology under different erosion conditions.

(2) The tensile strength of GFRP strips is extremely sensitive to the changes in erosion angle, velocity, time, and sanding rate. Specifically, the strength decreases by approximately 16.8% at an erosion angle of 90°, 28% at an erosion velocity of 31 m/s, and 35% after an erosion time of 50 min. The strength reduction caused by the sand flow rate saturates at about 6% and shows little further change once the sand flow rate exceeds 45 g/min. The effect of these factors on the elastic modulus of the material is relatively slight; the maximum drop is only about 6%.

(3) Digital image correlation analysis shows that the strain distribution in the erosion region is relatively uniform at low load levels. With the increase in tensile load, the strain value in the center region of erosion increases significantly and is much higher than that in the adjacent regions. Regardless of the increase in erosion angle, velocity, sand rate, or time, it leads to more serious deformation damage in the erosion center region, which makes it the first to be deformed and destroyed in the tensile process.

(4) The strain maps further confirm that the inhomogeneity of material deformation increases at higher erosion parameters. As the load level increases, the center region of the erosion shows an obvious stress concentration phenomenon, where the damage continues to accumulate and reaches the most serious degree, and eventually becomes the starting point of the overall failure of the material.

(5) A strain-data-driven factor-wise inverse identification approach based on interpolation and particle swarm optimization (PSO) was established. The inverse model reproduced the calibration conditions with a mean in-sample error of approximately 4.2%, whereas leave-one-condition-out validation yielded an overall mean error of 31.8%. Therefore, the present method should be regarded as a factor-wise inverse identification proof of concept with limited generalization to unseen conditions, rather than as a validated predictor for arbitrary coupled or field erosion conditions. Further development requires an expanded experimental database, independent validation, and additional long-duration and field-relevant erosion data.

## Figures and Tables

**Table 1:** Material physical parameters.

Material	*f_t_*/MPa	*E_f_*/MPa	*t_f_*/mm	*m_f_*/(g∙m^−2^)
GFRP	1680	7.2 × 10^4^	0.178	450
Epoxy resin	38	2.4 × 10^3^	—	—

Notes: *f_t_*: Tensile strength of glass fiber-reinforced polymer (GFRP) and epoxy resin; *E_f_*: Elastic modulus of GFRP and epoxy resin; *t_f_*: Single layer thickness of fiber cloth; *m_f_*: Mass per unit area of fiber cloth.

**Table 2:** Specimen grouping.

Code	Angle/°	Velocity/(m/s)	Sand Flow Rates/(g/min)	Time/min	No. of Specimens
1	15,30,45,60,75,90	26	55	30	18
2	90	16,19,23,26,31	55	30	15
3	90	26	15,25,35,45,55	30	15
4	90	26	55	10,20,30,40,50	15

**Table 3:** Strain figures of GFRP strips under different erosion angles.

Erosion Angle/°	0.2P_u_	0.4P_u_	0.6P_u_	0.8P_u_	P_u_
Control	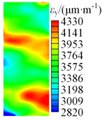	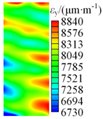	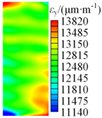	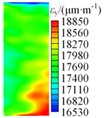	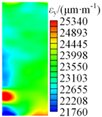
15	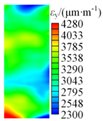	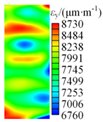	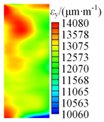	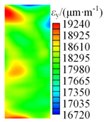	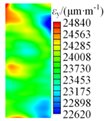
30	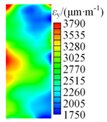	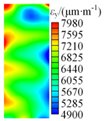	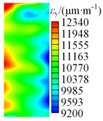	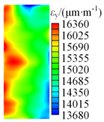	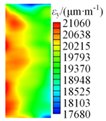
45	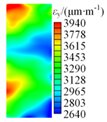	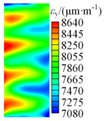	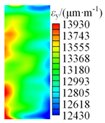	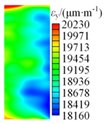	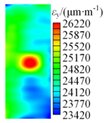
60	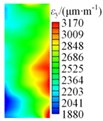	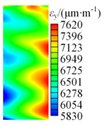	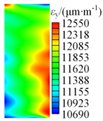	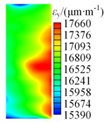	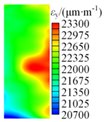
75	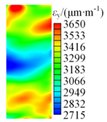	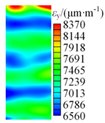	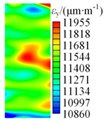	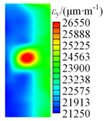	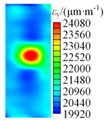
90	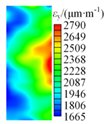	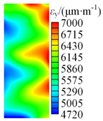	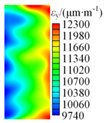	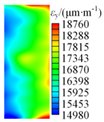	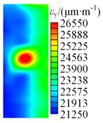

**Table 4:** Strain figures of GFRP strips under different erosion velocities.

Erosion Velocity/(m/s)	0.2P_u_	0.4P_u_	0.6P_u_	0.8P_u_	P_u_
16	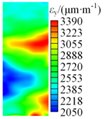	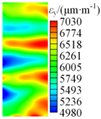	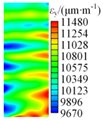	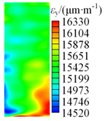	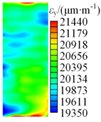
19	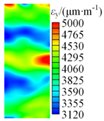	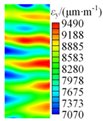	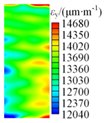	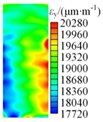	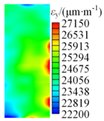
23	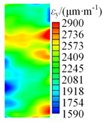	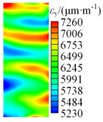	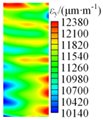	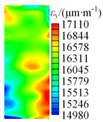	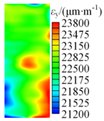
26	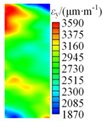	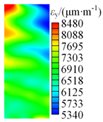	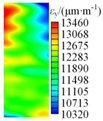	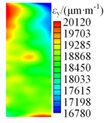	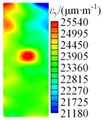
31	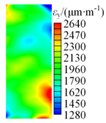	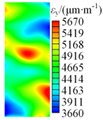	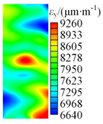	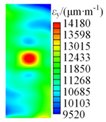	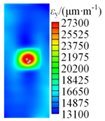

**Table 5:** Strain figures of GFRP strips under different sand flow rates.

Sand Flow Rates/(g/min)	0.2P_u_	0.4P_u_	0.6P_u_	0.8P_u_	P_u_
15	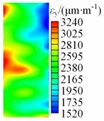	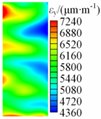	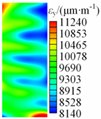	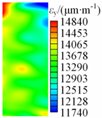	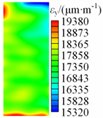
25	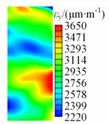	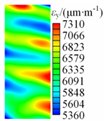	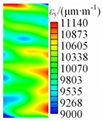	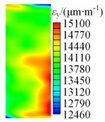	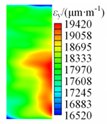
35	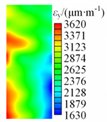	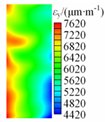	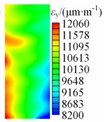	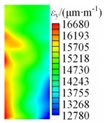	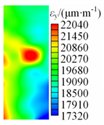
45	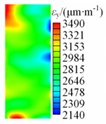	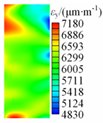	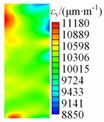	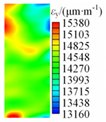	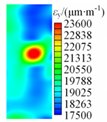
55	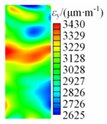	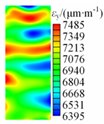	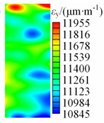	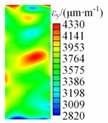	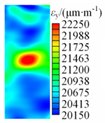

**Table 6:** Strain figures of GFRP strips under different erosion times.

Erosion Time/min	0.2P_u_	0.4P_u_	0.6P_u_	0.8P_u_	P_u_
10	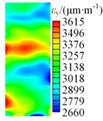	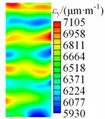	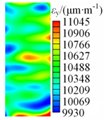	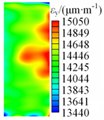	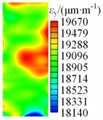
20	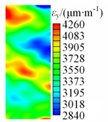	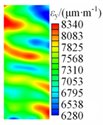	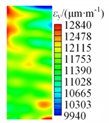	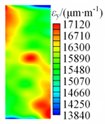	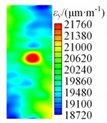
30	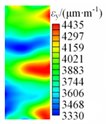	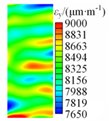	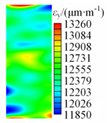	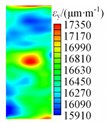	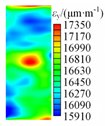
40	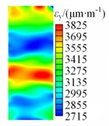	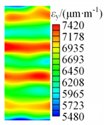	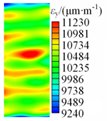	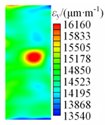	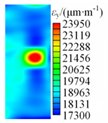
50	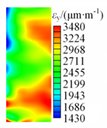	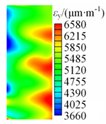	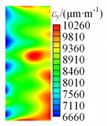	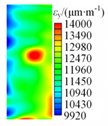	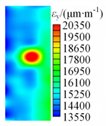

**Table 7:** Error analysis of the inverse identification model: in-sample versus independent (leave-one-condition-out) cross-validation.

Erosion Factor	Tested Range	In-Sample Mean Error	Held-Out Mean Error
Erosion angle	15–90°	6.1%	43.8%
Erosion velocity	16–31 m/s	4.8%	33.1%
Sand flow rate	15–55 g/min	5.7%	37.4%
Erosion time	10–50 min	0.4%	12.8%
**Overall**	-	**4.2%**	**31.8%**

## Data Availability

The original contributions presented in this study are included in the article. Further inquiries can be directed to the corresponding author.
